# Machine learning to predict genotypes and genotype-environment interaction associated with complex traits for genomic selection

**DOI:** 10.1016/j.plaphe.2026.100224

**Published:** 2026-05-19

**Authors:** Penghao Wang, Xiao-Qi Zhang, Viet Dang, Meixue Zhou, Sergey Shabala, Tianhua He, Chengdao Li

**Affiliations:** a Western Crop Genetics Alliance, Agricultural Sciences, College of Science, Health, Engineering and Education, Murdoch University, Murdoch, WA, 6150, Australia; b School of Molecular, Medical, and Forensic Sciences, Murdoch University, Murdoch, WA, 6150, Australia; c Cooper Medical School of Rowan University, Camden, NJ, 08103, United States of America; d Agriculture and Food, Department of Primary Industries and Regional Development, South Perth, WA, 6151, Australia; e Tasmanian Institute of Agriculture, University of Tasmania, Private Bag 1375, Prospect, TAS, 7250, Australia

**Keywords:** Genomic selection, Machine learning, De novo breeding, Haplotype prediction

## Abstract

Genomic selection (GS) can accelerate crop breeding and enhance selection efficiency. However, accurately predicting genomic estimated breeding values (GEBVs) for complex traits and applying GS in diverse environments remains challenging. To address these issues, we developed a novel hybrid method capable of modelling gene-gene and gene-environment interactions. This method offers precise predictions of phenotypic performance for complex traits, identifies haplotypes associated with desirable phenotypes, and enables prediction of optimal haplotypes tailored to specific environments. We evaluated the approach using a dataset of 855 barley lines, with phenotypic data for grain yield and flowering time collected across multiple environments. The model incorporated 30,543 SNPs, nine soil parameters, and six daily environmental variables, achieving high prediction accuracies, with correlation coefficients of 0.93 for flowering time and 0.82 for grain yield. Our method identified 10 haplotype blocks significantly associated with flowering time and 13 blocks with grain yield, collectively accounting for over 90% of the total genetic variance. Additionally, we predicted the phenotypic effects of each haplotype and identified elite varieties carrying the most favourable haplotypes for crossing design and selection. The method also allows prediction of untested genotype × environment combinations, enabling selection of optimal genotypes for targeted environments. To facilitate its application, we developed a web-based interface (accessible at [https://penghaowang.shinyapps.io/shinygui/]), which enables breeders to identify optimal haplotypes and the varieties that carry them, streamlining the process of haplotype-based, environment-informed breeding. We note that the reverse prediction framework is currently applied on a single-trait basis and does not resolve multi-trait trade-offs such as between flowering time and yield, which remains a topic for future extensions.

## 1 Introduction

The global human population is projected to surpass 9.9 billion by 2050, necessitating a 70% increase in food production to meet the rising demand [1]. Increasing incomes in developing countries have shifted dietary preferences towards a more balanced consumption of proteins, carbohydrates, lipids, and micronutrients, intensifying pressure on the global food supply [2]. This demand is worsened by the adverse effects of climate change, which reduce the yields of most major crops [[3], [4], [5]]. Addressing these unprecedented challenges requires the swift development of new crop varieties that are both high-yielding and resilient to climate stress.

Integrating technologies has driven crop improvement toward global food security [6,7]. Conventional crop breeding, which relies on phenotypic selection and backcrossing, is inherently time-consuming and limited in scope [8]. Advances in molecular marker technologies have expanded the genetic variation available to breeders and facilitated the discovery of quantitative trait loci (QTLs) [9,10]. This progress has laid the foundation for Marker-Assisted Selection (MAS), which has proven effective for simple traits governed by a few genes. However, MAS is inadequate for complex traits controlled by numerous small-effect loci [11]. The integration of genome-wide sequencing and precise phenotyping enables the calculation of genomic estimated breeding values (GEBVs), helping breeders cost-effectively identify breeding candidates. Leveraging GEBVs within the genome-wide prediction framework can accelerate genetic gain in breeding [12]. The advancement of sequencing technologies enables genomic selection (GS), which combines whole-genome molecular markers and phenotypic data to derive GEBVs, thereby achieving a more comprehensive and reliable selection [13].

In GS, a prediction model for phenotypic traits is constructed from a training population; thus, the genetic effects of unobserved individuals are predicted, avoiding the omission of small-effect genes that would fail to reach significance in typical genome-wide association studies [14]. GS calculates the GEBV for each line using genome-wide marker profiling, facilitating line selection prior to field phenotyping and effectively shortening the breeding cycle [15]. Since the selection is no longer constrained to traits determined by a few major genes, GS offers a promising avenue for genomic-assisted breeding and is significantly more efficient than MAS breeding and conventional pedigree breeding [7].

Current GS methods are broadly categorised into parametric statistical models and machine learning (ML) based approaches. Several factors constrain the performance of these parametric methods. First, the number of genomic markers considered in the model is much larger than the training population size, leading to high collinearity and making it challenging to estimate marker effects accurately, even with penalised regression and variable selection. Second, within the parametric framework, non-additive genetic effects are excluded from the model [16,17]. Third, genomic predictions were initially designed to model genotype-to-phenotype relationships under a single environmental condition; current parametric models struggle to effectively model site-specific environmental effects due to our limited understanding of the interplay between genomic variations and environmental factors, leading to unsatisfactory accuracy under genotype × environment interaction (G × E) conditions [13,16,17]. Fourth, conventional GBLUP and parametric models often perform poorly when predicting phenotypic performance (or genomic-estimated breeding values adjusted for environment) in environments not represented in the training set [18]. Machine learning approaches offer a compelling alternative, as they can inherently model complex G × G and G × E interactions without predefined biological assumptions [18]. ML models such as Support Vector Machines (SVM) [19], Reproducing Kernel Hilbert Spaces (RKHS) [20], Random Forests (RF) [21], Artificial Neural Networks (ANNs) [[22], [23], [24]], and Deep Learning [[25], [26], [27], [28]] have demonstrated improved prediction of G × E outcomes. Grinberg et al. showed that, while parametric models perform well for simple traits, ML methods outperform them for complex traits [29]. RF is the most robust in handling noise and missing data. Recent empirical research further indicates that ML methods can accurately predict phenotypic outcomes resulting from different combinations of genotypes, environments, and management interventions [30].

Despite advances in GS, most genomic-selection models remain limited to predicting phenotypic performance (or breeding values) from existing genotypes. These models are not designed to predict or generate new genotypes or haplotype combinations that would deliver a specified target phenotype under environmental or soil conditions [[31], [32], [33]]. This restricts their utility for breeding programs that aim for site-specific variety design, where the goal is not just to select among existing germplasm, but to identify or design genotypes optimised for a given environment and trait. Haplotype-based breeding, as proposed in Ref. [34], offers a practical solution by utilising blocks of linked genetic variation inherited together, thereby simplifying selection and crossing.

In this study, we present a novel GS method that incorporates G × E interactions along with detailed environmental and soil parameters to enable accurate phenotype prediction and haplotype identification. Our model also enables reverse prediction: identifying the optimal genotypic composition for any desired trait profile in a given environment, including those not observed during model training. We validated this approach using 855 barley lines genotyped with 30,543 SNPs and phenotypic data collected over two years from five geographically distinct sites across Western Australia. By integrating nine soil properties and six daily environmental variables throughout the growing season, our model achieved high predictive accuracy and identified key haplotype blocks associated with grain yield and flowering time. We developed a web-based tool (https://penghaowang.shinyapps.io/shinygui/) that allows breeders to explore optimal genotype-environment combinations and identify elite varieties carrying beneficial haplotypes. The term “elite haplotypes” refers to specific haplotype combinations within a haplotype block that are associated with favourable trait values (e.g., higher grain yield) within the evaluated panel. We refer to “optimal genotypes” as the set of haplotype profiles predicted to achieve a desired phenotypic target under a given environmental scenario. This platform supports haplotype-informed selection and crossing, streamlining the breeding pipeline for climate-resilient, high-performing crop varieties.

## 2 Materials and methods

(1) Genotype and phenotype data

We developed and evaluated our models using barley genotype data generated at the Western Crop Genetics Alliance (WCGA) at Murdoch University, as initially described in Refs. [32,35]. Briefly, 855 barley accessions, including breeding lines, cultivars, and landraces, were genotyped. These lines represent genetic diversity from 37 countries across all agricultural continents. After quality filtering based on heterozygosity, mapping quality (≥20), and a minor allele frequency (MAF) threshold of 0.01, a total of 30,543 high-quality single nucleotide polymorphism (SNP) markers were retained. The final dataset provided a genome-wide marker density of approximately one SNP per 150 kb.

Phenotypic data for the barley accessions were collected across five geographically distinct locations in Western Australia, spanning 12 large-scale field trials conducted in 2015 and 2016, as initially described in Refs. [32,33]. The trial sites represent diverse climatic conditions and are situated in separate agricultural zones (Ag Zones) (Fig. S1). Ten trials were conducted under natural light and rainfed conditions. In comparison, two trials included management treatments: one trial at South Perth in 2016 was supplemented with an extended photoperiod (18 h of light), while another at Merredin (Western Australia) included irrigation.

Our modelling centred on two key agronomic traits: grain yield, measured in kg/ha, and flowering time, recorded as the number of days to Zadok's stage 49 (ZS49) [36]. Detailed information on all experimental lines, including their genotypes, phenotypic, and environmental data, is available at https://doi.org/10.60867/00000010, https://doi.org/10.60867/00000003, and https://doi.org/10.60867/00000011, respectively.

(2) Environment data

We integrated environmental data from three distinct sources to comprehensively model the complex G × E interactions across our field trials.

Weather data from the nearest official stations: For each trial site, we identified the nearest meteorological station (details in Table S1) from publicly available Bureau of Meteorology (BOM) data. From sowing until harvest, we retrieved daily values of: minimum temperature (°C), maximum temperature (°C), the temperature range (difference between maximum and minimum, °C), rainfall (mm), and daily solar exposure (MJ m^−2^). In our study the environmental covariates (daily weather variables) were computed using actual sowing and harvest dates recorded for each trial. In practical breeding applications, such growing period boundaries are typically not known in advance and must be inferred or estimated from observational data or agronomic records (e.g., remote sensing, in-field phenology logging). As a result, the environmental descriptors used here represent an idealised case with known growing periods, and prediction accuracies may be optimistic relative to scenarios in which growing periods are uncertain.

In-field microclimate measurements: At each trial site in 2016, we deployed data-logging sensors (Tinytag Data Loggers, Omni Instruments, Dundee, UK) at two heights: approximately 20 cm above the ground (below canopy) and at canopy level. These sensors recorded air temperature (°C) and relative humidity (%) at 15-min intervals throughout the growing season; these high-resolution data provide a more accurate description of the microenvironment experienced by the plants.

Soil physical and chemical properties: We obtained site-specific soil data from the CSIRO Soil and Landscape Grid of Australia, accessible via data.csiro.au. For each trial location, we extracted nine soil attributes: available water-holding capacity in volumetric terms (AWC), whole-earth bulk density (BDw), fine-earth bulk density (BDf), clay content (CLY), proportion of coarse fragments (CFG), soil electrical conductivity (ECD), soil pH in water (pHw), sand content (SND) and silt content (SLT).

Management or intervention: To account for non-climatic environmental variation, we encoded site-specific treatments as categorical environmental covariates: the extended photoperiod (lighting) treatment at the South Perth site and the additional irrigation treatment at the Merredin site.

Finally, by combining these sources — daily weather, high-frequency in-field climate, detailed soil attributes, and management flags — we assembled a rich set of environmental variables for each trial. These variables were incorporated into our statistical modelling framework for G × E, providing both a broad-scale climatic context and a fine-scale representation of the field microenvironment and soil conditions.

(3) Exploratory and phylogenetic analysis

Exploratory and phylogenetic analyses were first performed to assess the genetic and phenotypic diversity of the dataset. A genetic distance matrix was calculated using the SNP genotypes of the 855 barley lines via the SNPRelate v1.22.0 package [37]. Principal component analysis (PCA) was conducted to examine the distribution of genotypic variation. The relationship between genotype and phenotype (flowering time and grain yield) was visualised using ggplot2 v3.3.3 [38]. To infer phylogenetic relationships, a Neighbour-Joining (NJ) tree was constructed using the ape v5.4-1 package [39] and visualised with ggtree v2.2.4 [40]. All analyses were performed in R v4.0.2 (R Core Team, 2021) using custom in-house scripts.

(4) Phenotype prediction

In this study we evaluated several genomic prediction models that differ in how they incorporate genotype-by-environment (G×E) interactions and environmental covariates. Throughout this manuscript, these models are referred to as: (i) “G”, a GBLUP baseline genomic best linear unbiased prediction ignoring explicit environmental variables; (ii) “G + E GBLUP”, a stratified environment GBLUP model including environment as a categorical factor; (iii) “G×E GBLUP”, a multi-environment model using reproducing kernel Hilbert space model to capture non-linear genetic effects and environment interactions; and (iv) “RF”, a random forest machine-learning approach incorporating genotype and environmental predictors.

(4.1) Baseline genomic prediction model (GBLUP)

Most parametric genomic-prediction models treat marker effects as random effects and can be formalised as:

(1) y=Xβ+Zα+ε,

where *y* is the vector of phenotypes; β represents non-genetic fixed effects (with a diffuse prior); *X* is the incidence matrix for fixed effects; *Z* is the *n*×*k* marker genotype matrix; α is the vector of random regression coefficients for all markers; and ε is the residual error. In ridge-regression BLUP, for example, α is assumed to follow a multivariate normal distribution with a common variance for all loci [41]. This hypothesis can be relaxed in multiple ways, including BayesA, BayesB, BayesC, and the least absolute shrinkage and selection operator (LASSO) approach [42].

A commonly used variant is Genomic Best Linear Unbiased Prediction (GBLUP), which replaces the explicit marker effects with a genomic relationship matrix (GRM) [43]. Under GBLUP, the genomic breeding value g=Zα has variance $Var\left(g\right)=G\;\sigma_{g}^{2}$, where *G* is the GRM derived from marker data. This formulation reduces the dimensionality of the problem and often improves prediction accuracy [[44], [45], [46]]. This baseline model uses only genomic information and does not account for environmental effects. In the Results and figures this model is labelled as “G”.

(4.2) Environment-stratified model (G + E GBLUP)

The G + E GBLUP model treats environment simply as a categorical factor and includes genotype and environment main effects but does not incorporate quantitative environmental covariates. This model appears in the Results and Figures labelled “G + E GBLUP”.

In genomic prediction, correctly modelling G × E (genotype × environment) interactions remains challenging. Traditional approaches typically either: (i) treat each environment separately by fitting marker effects independently within each environment (stratified-environment models), which seriously limits the ability to predict performance in new environments; or (ii) assume marker effects are identical across all environments (across-environment models), thereby ignoring environment-specific genetic interactions.

Formally, the G + E model can be expressed as:

(2) y=Xβ+Zu+We+ε,

where *y* is the vector of phenotypes; β represents fixed effects; *Z* is the incidence matrix for genotype effects; *u* represents genomic breeding values assumed to follow $u∼N\left(0,G\sigma_{g}^{2}\right)$; *W* is the incidence matrix for environment effects; *e* represents fixed environment effects corresponding to different trial environments; and ε is the residual error.

In this formulation, environmental differences among trials are captured through the categorical environment term *e*, while genomic relationships among genotypes are represented by the genomic relationship matrix *G*. However, because environments are represented only as categorical levels, this model does not explicitly incorporate quantitative environmental variables such as weather or soil descriptors. Consequently, although it captures overall differences among trial environments, it has limited ability to generalise predictions to new environments not observed in the training data. This model therefore serves as an intermediate benchmark between the baseline genomic model using genotype alone and the multi-environment G × E model that incorporates explicit environmental covariates.

(4.3) Multi-environment genomic prediction (G×E GBLUP)

(4.3.1) Model construction

To model genotype-by-environment (G×E) interactions while enabling prediction in unobserved environments, we adopted a reaction-norm framework following [46]. In this framework, phenotypes are modelled as the sum of main genetic effects, environmental effects, and their interaction, with both genomic and environmental information incorporated through covariance structures. The baseline reaction-norm model is:

(3) $$y_{ij}=\mu +g_{j}+w_{ij}+{gw}_{ij}+\varepsilon_{ij}$$

where: $y_{ij}$ is the phenotype of genotype j in environment i; μ is the overall mean; $g_{j}$ is the genomic effect of genotype j, with $g∼N\left(0,G\sigma_{g}^{2}\right)$, where *G* is the genomic relationship matrix derived from SNP markers; $w_{ij}$ represents the environmental effect; ${gw}_{ij}$ represents the genotype-by-environment interaction; and $\varepsilon_{ij}∼N\left(0,\sigma_{\varepsilon }^{2}\right)$ is the residual error.

In this formulation, the interaction term ${gw}_{ij}$ captures genotype-specific deviations across environments. Following [46], G×E is modelled through covariance structures rather than explicit marker-by-environment regression, which is infeasible in very high-dimensional settings.

(4.3.2) Incorporation of environmental covariates

Environmental effects were modelled using quantitative environmental covariates. Let W denote the matrix of environmental covariates (five daily weather variables: minimum temperature, maximum temperature, temperature range, rainfall, solar exposure; and nine soil properties), and γ the vector of regression coefficients. The environmental effect is defined as: $w_{ij}=W_{ij}\gamma$. The full model implemented in this study is therefore:

(4) $$y_{ij}=\mu +g_{j}+W_{ij}\gamma +{gw}_{ij}+\varepsilon_{ij}$$

In this formulation, $W_{ij}\gamma$ captures the fixed main effects of the measured environmental covariates, while the interaction term ${gw}_{ij}$ is modelled separately as a random effect. This separation allows the model to distinguish between overall environmental effects and genotype-specific responses to environmental variation, as well as enables prediction in new environments using their covariate profiles.

(4.3.3) Covariance structure of G×E interaction

The G×E interaction is modelled using covariance structures derived from genomic and environmental similarities. Let G be the genomic relationship matrix and Ω the environmental similarity matrix constructed from environmental covariates. The covariance of the interaction term is defined as: $\text{Cov}\left(gw\right)=\left(G\circ \Omega \right)\sigma_{gw}^{2}$, where ∘ denotes the Hadamard (element-wise) product.

This formulation implies that interaction effects are large when both genotypes and environments are similar. It avoids explicit estimation of all markers by environment interactions, and instead models G×E through covariance functions linking genomic and environmental similarity.

(4.3.4) Variable selection and dimension reduction

To limit model complexity and avoid overfitting, which occurs when the number of interaction coefficients increases rapidly with many environmental and genotypic predictors, we evaluated reduced-dimension representations of both environmental and genotypic data.

For the environmental variables, we compared three strategies: (1) temporal aggregation, in which daily weather data were summarised over a fixed 7-day interval, producing summary statistics such as total rainfall, average minimum and maximum temperature, solar exposure, and photoperiod duration for each interval from sowing to harvest; (2) automated variable selection, using caret's Recursive Feature Elimination (RFE) with a randomForest (RF) regressor (caret v6.0-86 package) and repeated five-fold cross-validation, to retain only those daily environmental covariates that contribute most to predictive accuracy; and (3) using the complete daily data set without reduction, to benchmark performance.

In RFE, the cross-validation folds were defined at the level of genotype-environment observations, such that all phenotype observations from a given genotype-environment combination remained together within a single fold. To avoid information leakage, the RFE feature-selection procedure was performed within the training folds of the cross-validation used for model evaluation.

Because the length of the growing period differed among trials and therefore the number of daily weather records varied by genotype-environment combination, we used the period covering all trial growing periods.

The RFE approach, by systematically removing less informative or redundant covariates, helps preserve key variables while reducing dimensionality and risk of overfitting. This comparison allowed us to identify the optimal balance between environmental resolution and model parsimony for accurate G×E prediction.

Soil parameters (available water-holding capacity, bulk density, clay content, sand content, silt content, electrical conductivity, pH, coarse fragments) were treated as fixed across years at each trial site. No temporal variation in soil parameters was assumed, reflecting the use of soil-grid data compiled at the site level.

After performing RFE with repeated cross-validation, we recorded the frequency with which each environmental and soil variable was retained in the reduced set across all repeats. Variables that were frequently retained (e.g., present in >70% of folds) were considered key predictors and examined for biological relevance. We subsequently report these selected features in the Results.

There is no missing weather or soil data for any of the trial sites. Missing genotypes are imputed by using the Wright method via the snpReady package version 0.9.6 [47]. A small number of missing phenotypic observations are removed before model fitting.

(4.3.5) RKHS implementation of the GxE model

The model in Eq. (4) was implemented using a Reproducing Kernel Hilbert Space (RKHS) framework to capture potential nonlinear genetic effects. In this implementation, the linear genomic relationship matrix *G* is replaced by a nonlinear kernel matrix *K* computed using a Gaussian kernel based on SNP markers.

Environmental covariates were not modelled using RKHS but were included as fixed effects (Wγ). The G×E interaction was modelled using an interaction kernel constructed as the Hadamard product of the genomic kernel *K* and the environmental similarity matrix. This provides a nonlinear extension of the genomic component while preserving the same reaction-norm model structure. The model was fitted using the BGLR R package (version 1.0.8), which implements Bayesian regression models with RKHS priors.

(4.4) Machine learning model (Random Forest)

The random forest model labelled “RF” in the Results section is a non-parametric machine-learning approach that incorporates all available SNP markers and environmental covariates as predictors, capturing interactions implicitly.

The RF model was implemented using the R packages randomForestSRC (v2.11.0) [48] and caret, with parallel processing via the doParallel package (v1.0.16). In the multivariate RF framework, all SNP markers and environmental covariates (and their interactions) were included as predictors; the final prediction is the average across 1000 decision trees, each trained on bootstrap resamples of the data, with hyperparameter tuning and selection based on root-mean-square error (RMSE).

(4.5) Model evaluation and cross-validation

All models were evaluated using ten repeats of five-fold cross-validation. In these cross-validation experiments, the data were split at the level of genotype-environment combinations: each fold contained a subset of genotype-environment pairs, such that the same genotype appearing in multiple environments could occur in both training and test folds but not the same observation.

This scheme evaluates how well the model predicts phenotypes for new genotype-environment combinations drawn from the same trial set. To assess generalisation to entirely new environments, we also employed a leave-one-environment-out scheme: data from one trial site (all genotype-environment combinations at that site) were excluded from training and used only for validation, simulating prediction in previously untested environments.

(5) Genotype prediction

**Fig. 1:**
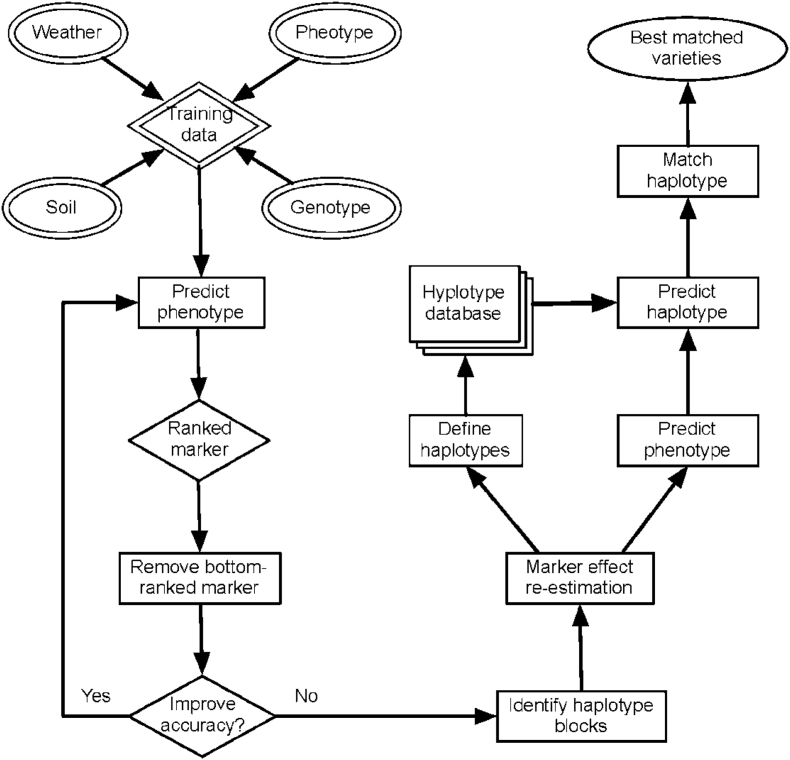
The overview workflow of the multi-step genotype and haplotype prediction algorithm.

While phenotype prediction is valuable in breeding applications, predicting genotypes that confer a desired phenotype in a specific environment poses a greater challenge than predicting phenotypes from known genotypes and environmental conditions. This difficulty primarily arises from two issues: the limited number of available samples relative to the number of predictors, and the imbalance between the number of predictors and the number of response variables. To address this, rather than attempting to predict the complete genotype across all markers, we focused on identifying key haplotype blocks, regions defined by clusters of closely linked SNPs, that were consistently associated with phenotypic variation. We selected these haplotype blocks based on their significant contributions to trait variation, as determined by our model. We implemented a multi-step selection procedure built on a multivariate RF approach to identify and prioritise these key haplotypes. An overview of the workflow is presented in Fig. 1.

(5.1) Identification of informative SNP markers

In the first step, we identified significant SNP markers associated with each target trait using a phenotype modelling procedure. All SNP markers were initially subjected to the RF-based RFE algorithm, which was consistent with the method used in our phenotype prediction model. Environmental factors and soil attributes were included as separate predictors during training. Using ten repetitions of five-fold cross-validation, SNP markers were recursively ranked according to their contribution to prediction accuracy for flowering time and grain yield. In this RFE process, cross-validation folds were defined at the level of genotype-environment combinations, identical to the scheme used in phenotype prediction cross-validation. That is, when ranking markers, each fold contained unique genotype-environment pairs to ensure that selection performance was evaluated on held-out observations rather than fragmented splits within the same combination. In each iteration of the RFE process, the lowest-ranked marker was removed from the model, and the RF was reconstructed. If excluding a marker improved prediction accuracy, the next lowest-ranked marker was removed. This process continued until a subset of approximately 150 top-performing markers was identified, optimising prediction accuracy for both traits. To account for potential modelling uncertainty and to support the downstream identification of key haplotype blocks, we extended this selection to include the top 200 SNP markers.

(5.2) Haplotype block construction

To identify haplotype blocks from the top-ranked SNP markers, we first extracted the 200 highest-ranking SNPs from the recursive feature elimination (RFE) procedure. We then scanned the physical positions of these SNPs along each chromosome. Any set of two or more top SNPs whose genomic positions lay within a 1.5 Mb window (i.e., the distance between the first and last SNP in the set ≤1.5 Mb) was considered as a candidate haplotype block. The 1.5 Mb threshold was based on prior analyses of linkage disequilibrium (LD) decay in our barley panel (following [49]) and reflects the typical distance over which LD remains sufficiently high for this material. We did not perform computational phasing; instead, blocks were defined solely based on physical proximity.

(5.3) Haplotype effect estimation

Once blocks were determined, we considered all possible allele combinations across the SNPs in each block (i.e., haplotype combinations), and using the multi-environment Bayesian GBLUP model, estimated individual SNP effects. We then calculated haplotype effects as either (i) the sum of individual marker effects (quantitative scheme) or (ii) using a simple signed scheme (alleles with positive effects assigned +1, those with adverse effects −1, heterozygotes half weight). We excluded blocks that contained fewer than two selected (top-ranked) SNPs, since such blocks have only one marker and therefore cannot define different haplotype combinations with meaningful variation. Finally, all 855 barley lines were indexed at each haplotype block according to the effect of the haplotype they carried, enabling downstream analysis for breeding decisions.

(5.4) Reverse prediction of optimal haplotypes

In this final step, we attempted to predict candidate haplotype profiles that would correspond to a desired phenotype under specified environmental and soil conditions; this constitutes a “reverse-prediction” from phenotype + environment to genotype (haplotype), a novel element of our method. We defined a desired phenotype as a target value (or range) for one of our focal traits, for example, earlier flowering or higher grain yield under a given environmental scenario (i.e., defined by the set of environmental and soil covariates characterising a trial site). For each environment in our dataset, we paired the observed value of the phenotype for a given line with that environment's covariates to form a “phenotype + environment” feature vector. When we invoked the model for design purposes, i.e., target selection, we specified a hypothetical desired phenotype value under a specified environment as input features. The response variable was the haplotype profile of each line across the previously defined key haplotype blocks (as defined in Step 2). For a line, the haplotype profile is a vector whose elements represent, for each haplotype block, which haplotype combination (allele combination across SNPs) the line carries.

We used a multivariate random forest (RF) model implemented via the randomForestSRC R package. All available predictors, the target phenotype (or its target value), and the environmental and soil covariates for that environment were used to predict the haplotype response vector. The haplotype prediction was trained and evaluated for each trait, with no multi-trait optimisation. Hyperparameters, including the number of trees, node size, etc., were tuned using cross-validation, and the final model was built with 1000 trees. In the randomForestSRC implementation, “multivariate” refers to the ability to model more than one outcome variable simultaneously within the same forest model. In standard regression forests, a scalar response (one outcome) is predicted. In a multivariate forest, a vector of outcomes, for example, multiple haplotype block assignments, can be predicted jointly, with splitting rules (e.g., composite splitting) accounting for multiple dimensions of the response. In our phenotype prediction, the response is univariate (single trait), but in the haplotype prediction stage, the response is a vector of haplotype block values; here the multivariate RF treats these block assignments as a multidimensional outcome to be predicted jointly.

In the context of haplotype prediction, the multivariate random forest model is trained to predict a vector of haplotype block assignments for each line under a specified environment and target phenotype. In this setting, each element of the output vector corresponds to a discrete haplotype category (allelic combination at that block). Conceptually, the prediction problem can be seen as multidimensional categorical prediction, where the response dimension equals the number of haplotype blocks, and each block can take several possible haplotypes. In a multivariate random forest framework implemented via randomForestSRC, the model treats this as a multivariate response problem, meaning that multiple response variables, haplotype block assignments in this regard, are predicted jointly within a single forest. Each tree in the forest attempts to partition the predictor space (consisting of genotype markers, environmental covariates, and target phenotype) in ways that reduce the composite impurity across all response dimensions simultaneously. This approach leverages correlation among haplotype blocks, which are patterns of co-occurrence across blocks, to improve joint prediction accuracy. Fig. S11 illustrates this model where the input matrix of predictors (genotype, environment, and desired trait) is mapped by the RF ensemble to a haplotype vector, with predictions aggregated across decision trees.

Although the output vector has high nominal dimensionality, several factors mitigate the effective complexity of the prediction task. Firstly, the barley panel exhibits linkage disequilibrium (LD) and population structure, such that not all haplotype combinations are equally probable. Many blocks have correlated patterns, reducing the effective degrees of freedom the model must learn. Secondly, prior to haplotype assignment, we applied recursive feature elimination to identify markers most predictive of the phenotype-environment relationship. This prioritisation reduces noise and focuses the model on the most informative genomic features, which can improve stability and predictive performance. Thirdly, blocks often share genetic influences due to physical linkage or shared causal loci. The multivariate RF framework can exploit these correlations, making joint prediction easier than independent block predictions.

(5.5) Cross-validation procedure

To assess predictive performance and generalisation of the haplotype prediction model, we implemented a coherent cross-validation strategy that combined repeated folds with nested internal procedures. We used ten repeats of five-fold cross-validation applied at the level of genotype-environment combinations, such that each held-out fold contained complete haplotype profiles for a subset of lines, and all remaining lines formed the training set. In each outer fold of the cross-validation, a subset of barley lines was held out as the test set. All downstream operations, including haplotype block selection, environmental data summarisation, data preprocessing, and model training, were conducted solely within the corresponding training set. This prevents any information from the test set leaking into the model and avoids post-selection bias. Within each repeat, the model trained on the training set was evaluated on the held-out test lines. Prediction accuracy was defined as the proportion of correctly predicted haplotypes across all blocks and test lines, computed as:

$$\text{Accuracy}=\frac{\text{number}\;\text{of}\;\text{block}-\text{haplotype}\;\text{assignments}\;\text{correctly}\;\text{predicted}}{\text{total}\;\text{number}\;\text{of}\;\text{block}-\text{haplotype}\;\text{assignments}}\times 100\%\text{.}$$

For each held-out line and each haplotype block, we checked whether the haplotype predicted by the RF model matched exactly the true haplotype observed in that line. Repeating the cross-validation process across 10 different random fold splits provided a stable estimate of model performance.

Following the prediction of an optimal (target) haplotype profile for a desired phenotype-environment scenario, we searched among all 855 barley lines in our panel for those whose haplotype profiles exactly matched the predicted profile. If no exact match existed, we computed a haplotype similarity/distance metric between the predicted profile and each line's observed haplotype profile. Specifically, we used a Hamming distance-based metric: for each haplotype block, we compared the haplotype identifier (allelic combination); any difference contributed a unit increment to the distance. The overall haplotype distance between the predicted profile and a line was defined as the sum of block-wise differences across all blocks. Lines with the smallest cumulative distance were considered to carry the most similar haplotype. This procedure enables identification of the closest available germplasm to the theoretically optimal haplotype combination predicted for a target trait under a specified environment, thus informing breeding decisions. We have developed a web tool to evaluate our method at https://penghaowang.shinyapps.io/shinygui/.

## 3 Results

(1) Phenotypes, genotypes and environmental data

**Fig. 2:**
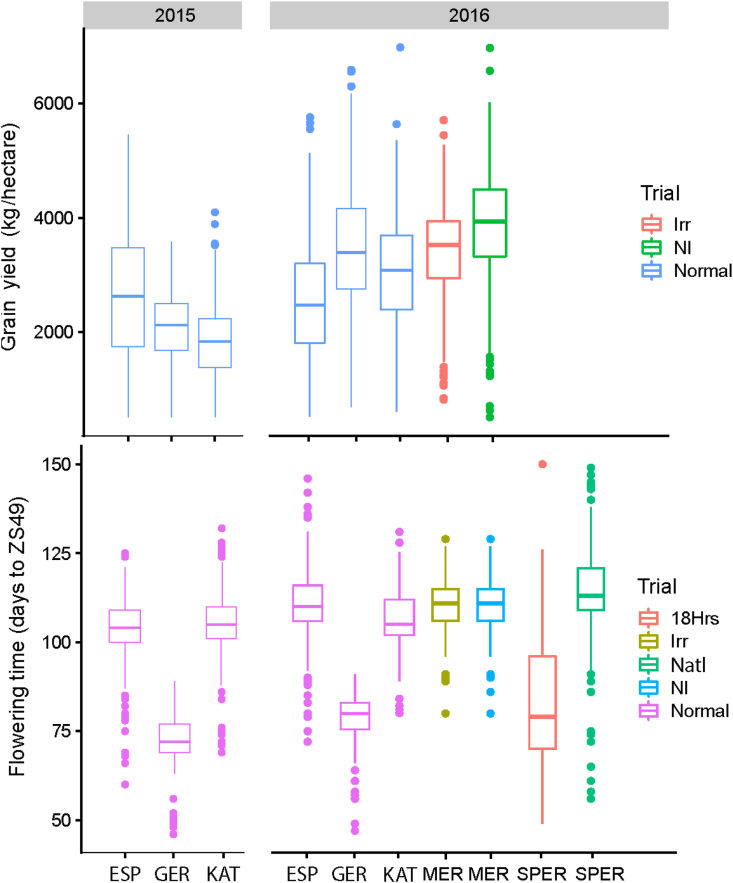
The flowering time and grain yield of the barley lines presented in the field trials. The subplot at the top shows the grain yield in kg per hectare, and the subplot below shows the days after planting to reach ZS49 as the flowering time. The abbreviations ESP, GER, MER, KAT, and SPER represent Esperance, Geraldton, Merredin, Katanning, and South Perth sites, respectively. The phenotypes observed at Merredin under irrigation treatment were marked as “Irr”, and those without irrigation were marked as “NI”. The phenotypes presented at South Perth under an 18-h’ artificial lighting experiment were marked as “18Hrs”, and those under natural lighting were marked as “Natl”. The other field trials were marked as “Normal”.

The flowering time (measured as days to ZS49) and grain yield of the 855 barley lines exhibited significant variation across the five trial sites and two growing seasons (Fig. 2). Barley lines grown in Geraldton flowered earlier than those in other locations, likely due to the warmer temperatures. Generally, flowering occurred earlier in 2015 than in 2016, with the most notable differences observed in Geraldton and Esperance. Under extended photoperiod treatments, barley lines tended to flower earlier than under natural lighting conditions (Fig. 2). Irrigation had a significant positive effect on grain yield at the Merredin site. These results highlight the critical influence of environmental factors on the phenotypic performance of the same variety. Genetic analysis revealed substantial diversity among the 855 barley lines (Figs. S2 and S3). However, this genetic clustering was not reflected in the distribution of phenotypic traits (Fig. S2), suggesting that G × E interactions play key roles in shaping phenotypic outcomes.

Environmental variables varied significantly across sites and years (Fig. S4–S9). Rainfall patterns were highly irregular, with isolated events, such as a three-day rainfall, contributing substantially to the total annual rainfall, particularly at the Geraldton site (Fig. S4). In contrast, solar exposure remained relatively consistent across all locations, gradually increasing from the beginning of the growing season into summer (Fig. S5). Temperature fluctuations were more pronounced. Minimum temperatures exhibited considerable day-to-day variability (Figs. S6 and S7), whereas maximum temperatures showed a general upward trend as the season progressed toward summer. Additionally, in-field sensor data, recorded at 15-min intervals, indicated significant intra-day fluctuations in both humidity and temperature, highlighting the fine-scale environmental dynamics captured during the growing season (Figs. S8 and S9).

(2) Phenotype prediction

**Fig. 3:**
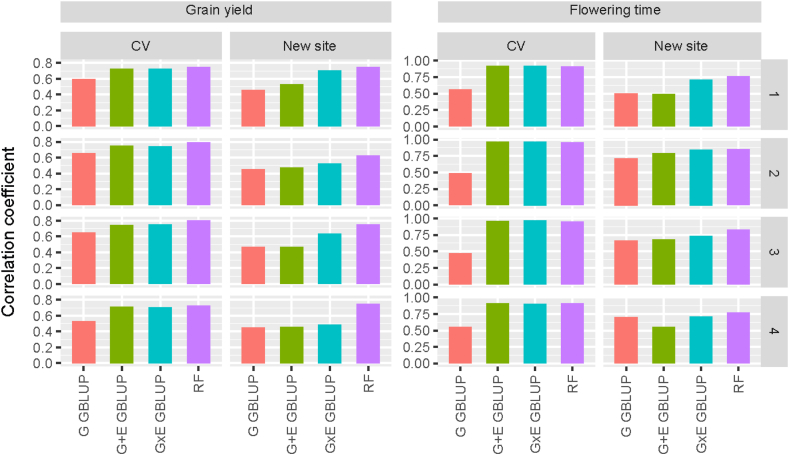
The left subplot shows the correlation between the predicted grain yields by the evaluated methods and the true ones, while the right subplot shows the correlation of predicted flowering times as measured in days from planting to ZS49 and the true ones. The “CV” vertical panels gave the results when models were trained using cross-validation on the whole dataset. The “New site” vertical panels display prediction results using one trial site as test data, which was not used for model training. The four horizontal panels correspond to the four evaluation strategies, with and without feature selection, for genotypes and environmental factors. “1” are the results when filtered markers and 7 days' average weather factors were used for modelling; “2” are the results when all markers and everyday weather factors were used; “3” are the results when filtered markers and all environmental factors were used; “4” are the ones when all the markers and 7 days' average environmental factors were used.

**Fig. 4:**
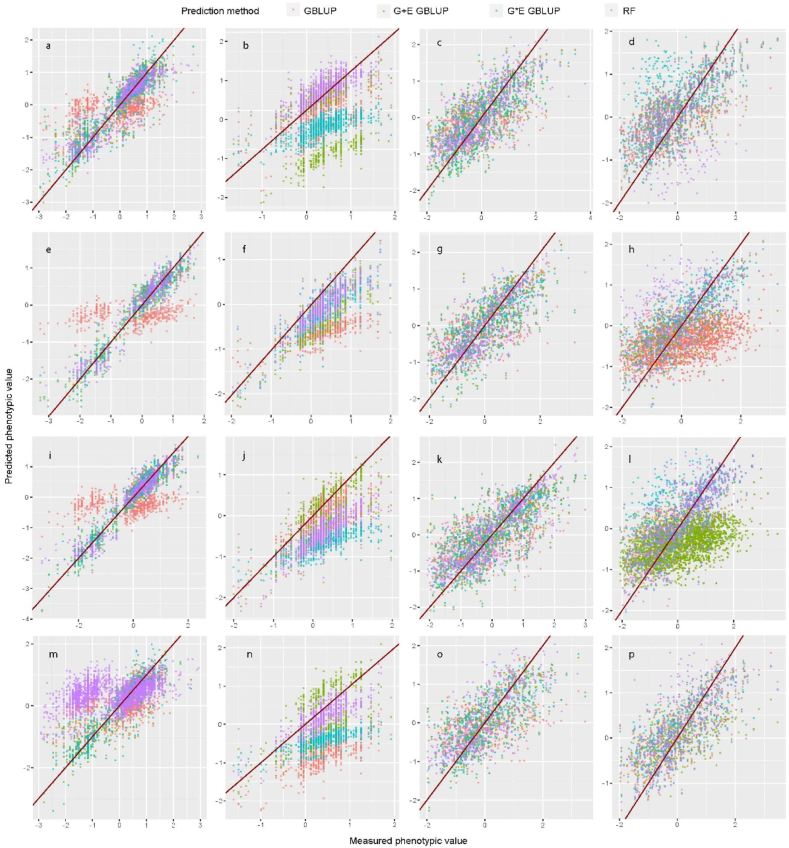
The correlations between the predicted flowering time and grain yield to the true ones. For all the subplots, the X-axis of the plots is the actual flowering time or grain yield, and the Y axis is the predicted ones. The red straight line is the 1-to-1 correspondence line. “GBLUP”, marked red, gives the result using the Bayesian GBLUP model using only genotypes; “G + E GBLUP”, marked green, is the environment-stratified Bayesian GBLUP method; “G∗E GBLUP”, marked blue, is the G x E RKHS model; and RF, marked purple, is the full G x E Random Forest-based ML prediction method. The subplots are organised as 4 by 4 grid. Each row of the plot represents one of the four evaluation strategies, and columns are flowering time and grain yield by cross-validation and unseen environment, respectively. (a), (b), (c), and (d) are the prediction performance of different methods using filtered markers and 7-day-averaged environmental factors. (a) shows the association between predicted ZS49 in days and the actual flowering time by cross-validation; (b) shows ZS49 prediction results on unseen environments where one trial site was left out of the model training and used as the testing dataset; (c) and (d) show prediction results of grain yield using cross-validation and unknown environments to the model, respectively. (e), (f), (g), and (h) are the prediction performance of different methods using all the markers and the day environmental factors. (e) shows the association between predicted ZS49 and real values by cross-validation; (f) shows ZS49 prediction results on unseen environments; (g) and (h) show the prediction results of grain yield using cross-validation and unknown environments to the model, respectively. (i), (j), (k) and (l) are the prediction performance using filtered markers and every day environmental factors. (i) shows results of ZS49 by cross-validation; (j) shows ZS49 prediction results on unseen environments; (k) and (l) show prediction results of grain yield using cross-validation and unknown environments, respectively. (m), (n), (o), and (p) are the results using all the markers and averaged environmental factors. (m) shows the results of the ZS49 prediction by cross-validation. (n) ZS49 prediction results on unseen environments. (o) and (p) show prediction results of grain yield using cross-validation and unknown environments, respectively.

Using the G × E RKHS and RF models, we predicted both flowering time and grain yield based on the genotypes of the barley lines, environmental conditions, and agronomic treatments (Fig. 3, where “GxE” represents GxE RKHS model, “RF” represents the RF model, and “G + E” represents the stratified environment model using GBLUP). The strength of the association between the model-predicted values and the observed phenotypes is illustrated in Fig. 4. When the actual quantified environmental variables and agronomic treatments were included in the models, our approach achieved a correlation coefficient of 0.93 for flowering time and 0.82 for grain yield. The G × E RKHS and RF models, by incorporating detailed environmental and management data, consistently outperformed both the traditional GBLUP model and the stratified environment approach, which treats environment and treatment conditions as a single categorical factor. These results highlight the advantage of explicitly modelling environmental variation to enhance the prediction of complex traits.

When G × E interactions were not modelled, our approaches achieved moderate prediction accuracies, with correlation coefficients of approximately 0.7 for flowering time and 0.5 for grain yield. However, including environmental effects, whether through the conventional stratified approach or a full G × E interaction model, led to substantial improvements in predictive performance. Specifically, correlations exceeding 0.9 were attained for flowering time and above 0.8 for grain yield. When the models were challenged to predict phenotypes in environments not included in the training set, the traditional method, which treats the environment as a single categorical variable, performed poorly, yielding accuracies similar to those of models that only consider genotype. In contrast, the RF and full G × E models, which incorporate quantitative environmental variables, maintained high predictive performance even under unseen environmental conditions (Fig. 3, Fig. 4). For flowering time, these models achieved correlation coefficients as high as 0.9, compared to 0.5–0.7 with the stratified approach. We also assessed the impact of dimensionality reduction by comparing model performance with all SNP markers to that with a subset of informative markers. For the flowering time, the RF model utilising all markers achieved a correlation of 0.96, compared to 0.93 using only the top-ranked markers identified through feature selection. Similar trends were noted for grain yield, with prediction accuracy improving from 0.7 to 0.82 when using the complete marker set (Fig. 3).

(3) Haplotype blocks and significant haplotypes

**Fig. 5:**
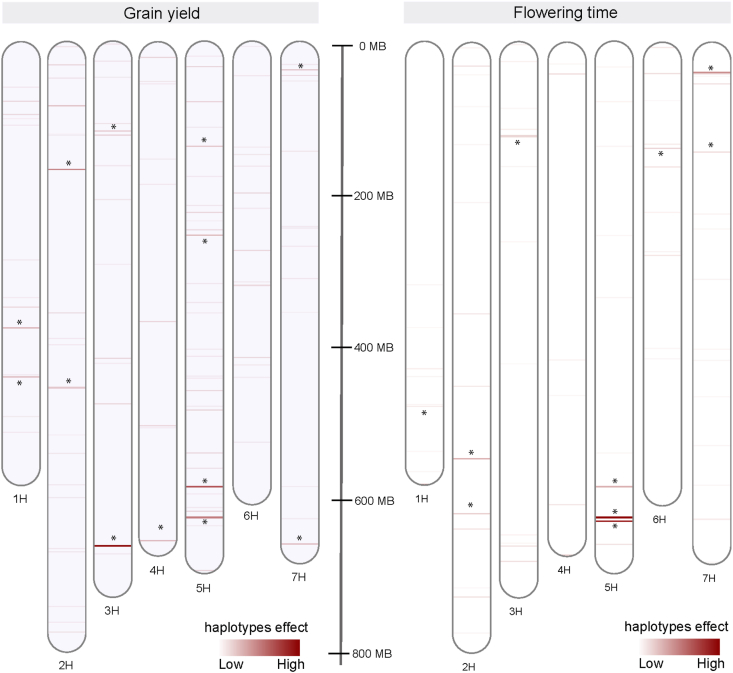
The location and effects of the identified haplotype blocks associated with flowering time and grain yield. The left subplot is the grain yield, while the right is the flowering time (days to ZS49). Each chromosome is represented by 1 bar and length relative to its size. The positions of haplotype blocks were marked as red lines on the chromosome bars, with the colour representing the effects of the haplotype. The darker the red, the more significant the effect. The size of the haplotype block is represented as the thickness of the lines. The top 13 haplotype blocks and top 10 flowering time haplotype blocks, which account for >90% of effects, were labelled by “∗”.

Our dimension-reduction procedure identified a subset of 200 top-ranked SNP markers that achieved prediction accuracy comparable to that of models using the complete genotype dataset. Using a LD decay distance of 1.5 Mb, we identified 84 haplotype blocks linked to flowering time and 92 blocks associated with grain yield based on these selected markers (Fig. 5). We identified 10 significant haplotype blocks (comprising 87 SNP markers) for flowering time and 13 blocks (comprising 60 SNP markers) for grain yield, which collectively accounted for over 90% of the total additive genetic variance on the respective traits. Across the 855 barley lines, we observed an average of 77 haplotypes per block (ranging from 31 to 131) in the 10 flowering time-associated blocks, and an average of 55 haplotypes per block (ranging from 18 to 158) in the 13 grain yield-associated blocks. Comprehensive details of the identified haplotype blocks, including constituent SNPs and their associated effects, are provided in Supplementary File 1 (flowering time) and Supplementary File 2 (grain yield).

Each haplotype block exhibited a distinct effect on the associated phenotype. By predicting the effects of every possible haplotype within each block, we identified both the best- and worst-performing haplotypes, which contributed to higher or lower grain yield and earlier or later flowering, respectively. Detailed results for grain yield and flowering time are provided in Supplementary Files 3 and 4. We extended the prediction framework to include haplotypes not observed in the original training population, allowing us to estimate their potential effects on flowering time and grain yield. Notably, in some haplotype blocks, the best-performing haplotypes were absent from all 855 barley lines. For instance, the haplotype block with the strongest effect on grain yield, defined by ten SNPs and located on chromosome 5H (designated as Hap_Chr5H_3), had an optimal haplotype that was not present in any of the evaluated lines. Interestingly, several benchmark Australian commercial varieties, including Hindmarsh, La Trobe, and Vlamingh, contained haplotypes in this block that negatively affected grain yield (Supplementary File 3). Despite this, we identified existing lines carrying the optimal haplotypes in eight of the 13 grain yield-associated haplotype blocks, offering immediate value for breeding programs focused on yield improvement (Supplementary File 3).

(4) Genotype prediction

**Fig. 6:**
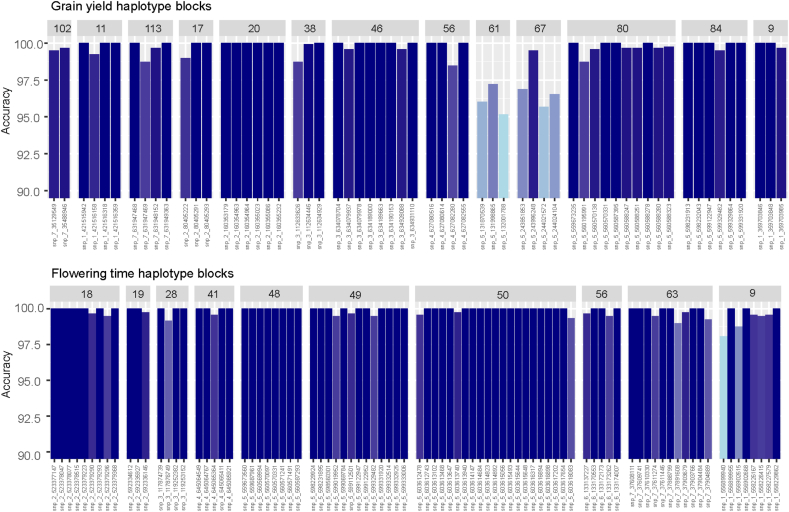
The haplotype/genotype prediction accuracy of our multistep RF method. The upper subplot shows grain yield, while the lower subplot shows flowering time. Each panel/grid in the subplot corresponds to a specific haplotype block. The averaged cross-validation prediction accuracy (the percentage of predicted markers that match the real marker exactly) is shown as a percentage, indicated by the height and colour of each bar. The number for each panel is the unique haplotype ID assigned by the algorithm.

We employed a multi-step genotype prediction approach (illustrated in Fig. 1) to assess haplotype performance. Our model exhibited high accuracy in predicting optimal haplotypes for grain yield and the latest flowering time (ZS49) under specific environmental conditions. Specifically, we achieved an average haplotype prediction accuracy of 99.0% for the latest flowering time and 97.1% for the best grain yield (Fig. 6). For all identified significant flowering time haplotype blocks, accuracies ranged from 98% to 100%. Similarly, all but two grain yield haplotype blocks had accuracies within this range; the exceptions had accuracies of 95%-98%, likely due to missing genotype data.

## 4 Discussion

Genomic prediction models were initially developed to clarify genotype-phenotype relationships in controlled, single-environment settings, aiming to minimise environmental variability within breeding trials and isolate genetic effects from environmental noise [13]. However, environmental factors are crucial determinants of agronomic traits, such as grain yield. Consequently, phenotypic expression arises from complex genotype-by-environment interactions unique to each environment. This study introduces a novel hybrid genomic prediction method that incorporates environmental data to capture these interactions better, thereby enhancing prediction accuracy across various environmental conditions.

In barley, temperature is a critical determinant of flowering time. Research indicates that specific genes, such as VRN-H1, VRN-H2, VRN-H3, PPD-H1, PPD-H2, and eam6/eps2, interact with environmental cues, such as temperature and photoperiod, to regulate phenological development. The allelic diversity and interactions of these genes significantly influence barley's adaptation to varying climatic conditions [32,33]. Similarly, in sorghum, environmental factors, particularly temperature, play a substantial role in phenotypic traits. Studies have shown that temperature variation across environments accounts for a significant portion of the variation in flowering time [50]. Regarding grain yield, rainfall is often a determining factor, especially in rainfed agricultural systems. In regions like Australia, where agriculture is predominantly rainfed, rainfall amount and distribution can significantly impact crop yields [51]. Given these insights, the accuracy and reliability of GS models are enhanced when site-specific environmental variables are incorporated. By accounting for environmental variables such as temperature and rainfall, GS models can more effectively predict phenotypic outcomes, improving crop performance and adaptation strategies.

Our prediction model, which combines Bayesian GBLUP with a Random Forest machine learning approach, effectively incorporates site-specific environmental information to predict complex trait phenotypes. The model exhibited strong predictive capability in both tested and untested environments. When environmental variables were included, it achieved correlation coefficients of 0.93 for flowering time and 0.82 for grain yield, the highest reported for these traits. These findings emphasise the importance of integrating site-specific environmental data into genomic prediction models. Our feature selection process identified several soil parameters, including water-holding capacity, sand content, and whole earth bulk density, as top predictors for both grain yield and flowering time. Moreover, incorporating daily weather variables surpassed growth-period averages, underlining the nuanced and stage-specific environmental influences on trait development. We examined the environmental and soil variables retained by our feature selection procedure to understand which factors most strongly contributed to prediction accuracy. The RFE approach consistently identified several soil attributes, including water-holding capacity, sand content, and whole-earth bulk density, as among the most informative predictors for both flowering time and grain yield predictions. In addition, daily minimum and maximum temperature and rainfall variables were frequently selected across cross-validation folds, underscoring their influence on phenotypic responses. The frequency with which each feature was selected is summarised in Supplementary Fig. S10, where variables are ranked by selection count across repeats of the selection procedure. We also note that because soil variables are fixed within sites across years, they may implicitly encode site identity, which can artificially inflate predictive performance in cross-site validation. In settings where soil profiles differ substantially or where new sites exhibit soil characteristics outside the range represented here, model transferability may be limited. Future work should evaluate the robustness of genomic prediction models under soil-variable perturbation or exclusion, for example by withholding sites with distinct soil profiles during validation or by explicitly testing models trained without soil covariates.

Our results align with recent studies [50,52,53], emphasising that parameterising environmental variables enhances model predictive power. Including genotype × environment × phenotype interactions in genomic prediction represents a crucial advance in integrating genomics, breeding, and agronomy and can significantly accelerate crop improvement [54]. By leveraging machine learning methods, we demonstrate the ability to capture complex, non-linear interactions that may not be well understood but are vital to prediction accuracy. This study demonstrates that incorporating quantitative daily weather and soil data enables accurate phenotype prediction across previously untested genotype-environment combinations, supporting more precise and adaptive breeding strategies. Because our model uses constant soil variables for each trial site, these variables may implicitly capture site identity, potentially inflating model performance when predicting across sites. This is a limitation to our model because predictions may not generalise to novel sites whose soil profiles differ considerably. Another limitation of our approach is that the temporal delineation of environmental windows (from sowing to harvest) was derived from trial records. In real-world breeding programs, phenological stages may not be known a priori and must be estimated, which could introduce additional uncertainty into environmental covariates and lower prediction accuracy compared to what was observed here. Future work could explore methods to estimate phenology or growing periods directly from observational data (e.g., crop monitoring, thermal time modelling), thereby making predictions more robust to unknown environments. Nevertheless, our method provides a viable strategy for extrapolating environmental parameters and edaphic profiles to support prediction. In practice, crop breeders often evaluate a large number of new breeding lines in a limited number of environments, followed by more extensive testing of a smaller subset of selected lines across a broader range of environmental conditions. Understanding the relationship between breeding values estimated in initial environments and those averaged across diverse environments is critical for designing efficient selection strategies. Instead of selecting breeding lines solely based on mean trait performance across environments, our model offers a more comprehensive and environment-specific approach. By parameterising environmental conditions within the G × E modelling framework, our method enables accurate prediction of phenotypic performance in untested environments, thereby supporting more informed and targeted selection decisions [18]. For instance, our model accurately predicted the specific haplotypes required to achieve a desired flowering time or grain yield under known and novel environmental conditions. These results demonstrate that incorporating quantified environmental descriptors into the model enables simulation of performance in environments not represented in the training data, thereby enhancing the predictive power and applicability of genomic selection in real-world breeding programs.

Our findings align with prior research emphasising the importance of integrating environmental and management variables into genomic prediction models to enhance predictive accuracy and adaptability to climate change [6,30]. Previously, de los Campos et al. developed a data-driven simulation platform that combines field trial data, genotype profiles, and historical weather records to predict cultivar performance under uncertain weather conditions [55]. This approach not only forecasts expected performance but also evaluates the distribution of a cultivar's performance across various potential weather scenarios, accounting for uncertainties in model parameters. Similarly, Washburn et al. [56] incorporated management practices into their models, demonstrating that convolutional neural networks (CNNs) can effectively predict agronomic yield when provided with comprehensive genetic, environmental, and management data. Their findings suggest that CNN models perform comparably to or better than standard genomic prediction methods, particularly when sufficient data across these domains are available. As climate change continues to alter agricultural production environments, utilising all available data resources, including genomic, environmental, and management information, is crucial for predicting crop performance in future scenarios [55]. Implementing these integrative approaches is vital for developing resilient agricultural systems, as they facilitate the rapid assessment of trait adaptability and stability, thereby supporting more effective breeding and management strategies [16].

GWAS have been crucial for identifying QTLs for key agronomic traits across different crops. However, the practical application of these findings in breeding programs is often constrained due to the large number of markers with effects are not clearly understood from a breeder's perspective. To tackle this challenge, haplotype-based breeding has emerged as a promising strategy. This approach focuses on identifying and utilising superior haplotypes, combinations of alleles at adjacent loci inherited together, to achieve desired phenotypic outcomes. By leveraging these “desired-phenotype-associated” haplotypes, breeders enable more precise parental selection and enhance genetic gains within breeding programs through the strategic rearrangement of haplotypes.

Our method enabled the identification of superior haplotypes associated with key agronomic traits. From the 84 marker-trait associations linked to flowering time, we identified 10 haplotype blocks that accounted for more than 90% of the total additive genetic variance. Similarly, 13 haplotype blocks out of 92 associations accounted for over 90% of the genetic variance in grain yield. A number of these haplotype blocks overlapped with previously known genes associated with the respective traits. For example, three closely linked haplotype blocks on chromosome 5H contained eight genes (HvCBF2A, HvCBF3, HvCBF10A, HvADA2, HvPhyC, HvBM5, HvAGLG1, and HvCK2A) previously identified in flowering time regulation [33]. Similarly, the 13 grain yield-associated haplotype blocks included several genes with known functions in yield determination, such as HvGARMP-2, HvGA20ox2, HvPI-2, HvCIGARP-2, HvCMF4, HvCBF4A, HvCBF14, HvCBF3, HvCBF10A, HvADA2, HvBM5 (HvVRN-H1), and HvAGLG1 [33]. We further identified the superior haplotypes within these blocks, along with the accessions that carry them (Supplementary Files 3 and 4). This information provides a powerful resource for genomic-assisted selection and enables targeted parental selection by identifying complementary favourable haplotypes. This allows breeders to intentionally assemble optimised haplotype combinations with predictable and desirable phenotypic outcomes, supporting the concept of haplotype-based breeding [34]. For instance, two haplotype blocks with the strongest effects on grain yield were identified on Chr3H (HB-Chr3H-634078704) and Chr5H (HB-Chr5H-559607323). HB-Chr3H-634078704 included 62 distinct haplotypes among the 855 barley lines, but only nine lines carried the top five haplotypes with significantly positive effects on grain yield (Supplementary File 3). Similarly, for HB-Chr5H-559607323, only four lines carried the top-performing haplotypes. In a practical breeding scenario aimed at improving the grain yield of an existing variety, our results show that only a limited number of crosses may be necessary to introgress high-performing haplotypes. Thus, our approach provides an efficient and scalable strategy for identifying optimal crossing candidates, enhancing the practical utility of genomic-assisted breeding. This study also contributes to a new paradigm in haplotype-based breeding, an approach with growing success in other crops such as pigeon pea [57], wheat [6], and maize [30], highlighting its broad potential for genetic improvement across species.

Unfortunately, we have no pedigree information for our barley panel, and reported predictive performance refers to within-panel accuracy. It may not represent performance in very distantly related or novel germplasms. In future work, to test generalisation more rigorously, we plan to perform “leave-population-out” validations by genetic similarity or test on independent, unrelated barley panels. Currently, our model can predict haplotypes for a single trait, and we plan to incorporate multiple-trait optimisation in the model as future work. It is important to clarify that the reported proportion of additive genetic variance (>90%) explained by selected haplotype blocks is conditional on the specific population, marker density, and haplotype definition strategy used in this study. This high proportion does not imply that a small number of genomic regions biologically “control” the traits in all contexts; rather, within this panel and modelling framework, those blocks capture a large share of the modelled additive variance. In other populations, or with different marker densities or block definitions, the set of influential regions and the proportion of variance they explain may differ substantially. Therefore, these results should be interpreted as descriptive of this dataset and modelling approach, not as universal biological determinants of trait architecture. We note that the haplotype-prediction framework presented here remains a computational and conceptual demonstration; actual deployment in breeding would require multi-generation crossing, phenotyping, and field trials under target environments, tasks beyond the scope of the present study. Consequently, the haplotype combinations identified should be regarded as candidates, not validated cultivars, and further empirical validation is needed before practical use.

In this study we report overall prediction accuracy: the proportion of correctly predicted haplotype assignments across all blocks and lines as the primary performance metric for the multivariate random forest model. We recognise that overall accuracy can be influenced by class imbalance when a small number of haplotypes dominates a block and may not fully reflect performance on rarer haplotypes. More detailed metrics such as precision, recall, F1 score, and Matthews Correlation Coefficient (MCC) can provide additional insights, but these require per-block and per-class evaluation and were beyond the scope of the current analysis due to time constraints and the multivariate nature of the prediction output. Despite these limitations, overall accuracy remains a reasonable measure of aggregate model performance when interpreted in context. Many haplotype blocks in our dataset have a limited number of high-frequency haplotypes and exhibit substantial linkage disequilibrium, which reduces the effective output complexity relative to the number of nominal blocks. We therefore use overall accuracy as a summary metric, but we emphasise that interpretation of Fig. 6 should consider the potential influence of class imbalance and that future work could incorporate additional block-wise performance metrics to characterise model behaviour in greater detail.

While our reverse prediction framework demonstrates strong performance within the barley panel studied here and across environments represented (at least partially) in the training data, we emphasise that this reflects within-panel prediction accuracy and interpolation across observed conditions. True extrapolation to entirely novel environments, with climatic or soil profiles not represented in the training set, and to genetically distant germplasm remains to be demonstrated. Such validations would require independent datasets collected under environmental conditions and genetic backgrounds distinct from those used in this study. We therefore constrain our claims to within-panel generalisation and note that broader applicability of the method should be tested in future work, for example through experiments on independent breeding panels or multi-year, multi-region trials with non-overlapping environments.

## 5 Conclusion

In recent decades, genomic selection has emerged as a powerful breeding tool that accelerates progress and enhances selection accuracy. The presence of genotype-environment-phenotype interactions in these crops necessitates their inclusion in any predictive models aimed at improving crop productivity. Genomic selection propels crop improvement by merging genomics, breeding, agronomy, and climate science. For instance, breeding climate-resilient crops requires forecasting performance in future environments that have not been encountered in tested populations. Integrating genomic selection with machine learning approaches can effectively meet these complex demands for future crop improvement. Our method also signals a shift from individual DNA markers to haplotype-based breeding, enabling future cultivar development by identifying and incorporating superior haplotypes into breeding populations. Our model, which combines RKHS GBLUP with a Random Forest ML procedure, accurately predicts phenotypic values of complex traits in barley and extends predictions to untested environments. Furthermore, our model identifies superior haplotypes that can be practically utilised for selecting parents in breeding crosses. Overall, our study highlights the potential of integrating genomic selection and haplotype-based breeding to release the full benefits of genomic-assisted breeding.

In recent decades, genomic selection has emerged as a transformative tool in crop breeding, offering the potential to accelerate genetic gain and enhance selection accuracy. Genotype-environment–phenotype interactions in many crops highlight the necessity of incorporating environmental parameters into predictive models to improve crop productivity. Genomic selection facilitates this integration by bridging genomics, breeding, agronomy, and climate science. To develop climate-resilient cultivars, it is essential to forecast performance in future, untested environments. When combined with machine learning, genomic selection can meet this challenge by modelling complex, non-linear interactions between genotypes and diverse environmental variables. Our approach illustrates this potential, representing a paradigm shift from individual markers to a more powerful haplotype-based breeding framework. The model we developed, which integrates RKHS GBLUP with an ML-based Random Forest algorithm, accurately predicts phenotypic performance for complex traits in barley, even under unseen environmental conditions. Furthermore, it enables the identification of superior haplotypes, providing breeders with a practical and efficient tool for parental selection and cross-design. Overall, our study demonstrates the strength of combining genomic selection with haplotype-based breeding, offering a robust strategy to unlock the full potential of genomic-assisted crop improvement.

Finally, we emphasise that the current implementation of the reverse prediction framework addresses one trait at a time; it does not perform multi-objective optimisation across traits and thus does not inherently resolve potential trade-offs between traits such as flowering time and grain yield.

## Author contributions:

CL, TH, and PW perceived the project concept; PW conducted modelling and data analysis; VD contributed to modelling; XZ, SS, and MZ collected materials and conducted phenotyping; TH and XZ conducted the genotyping; and PW, TH, SS, and CL wrote the paper with input from other authors.

## Funding

The Australia Grain Research and Development Corporation (DAW00240 and UMU00050).

## Declaration of competing interest

The authors declare that they have no known competing financial interests or personal relationships that could have appeared to influence the work reported in this paper.

## Data Availability

All the data and source codes have been uploaded to GitHub and can be accessed under the GNU Open License at: https://github.com/pwang2019/GxE_Model.

## Appendix A Supplementary data

Multimedia component 1

Supplementary File 1 The detailed list of the top 10 haplotype blocks and the top-ranking SNP markers associated with flowering time. Each row represents one SNP marker, and the column ID is the SNP ID, chrom is the chromosome, POS is the location of the marker, Hap_Block is the haplotype block ID that containing the SNP marker, Block_Start is the starting position of the haplotype block that contains the marker, Block_End is the end position of the haplotype blocks, Marker_Count is how many top-ranking SNP marker that haplotype block contains. TotalW is the total effect of the haplotype block on phenotype, calculated by summing the absolute effects of all contained markers. MeanW is the mean absolute effect of the haplotype block. Positive_effect is the if the marker has a positive effect on phenotype, +1 represents a positive effect while −1 represents a negative effect, and TotalB is the weighted effect on the phenotype when positive and negative effects are considered. Gene_ID is the annotated gene accession, and Gene_Name is the name of the gene. REF is the reference allele, which is the barley variety Morex, and ALT is the alternative allele. Columns afterwards are the genotypes of the barley lines.

Supplementary File 2 The detailed list of the top 13 haplotype blocks and the top-ranking SNP markers associated with grain yield. Each row represents one SNP marker, and the column ID is the SNP ID, chrom is the chromosome, POS is the location of the marker, Hap_Block is the haplotype block ID that containing the SNP marker, Block_Start is the starting position of the haplotype block that contains the marker, Block_End is the end position of the haplotype blocks, Marker_Count is how many top-ranking SNP marker that haplotype block contains. TotalW is the total effect of the haplotype block on phenotype calculated by summing the absolute effects of all contained markers, MeanW is the mean absolute effect of the haplotype block. Positive_effect is the if the marker has a positive effect on phenotype, +1 represents a positive effect while −1 represents a negative effect, and TotalB is the weighted effect on the phenotype when positive and negative effects are considered. Gene_ID is the annotated gene accession, and Gene_Name is the name of the gene. REF is the reference allele, which is Morex, and ALT is the alternative allele. Columns afterwards are the genotypes of the barley lines.

Supplementary File 3 All the predicted haplotypes for each of 13 key haplotype blocks and their mapping to all genotyped barley lines associated with grain yield. Each sheet of the Excel file represents one haplotype block. Hap is the predicted haplotype; Lines are the barley lines that have the same haplotype where each line is separated by “|”; Line_Count is the number of lines have the same haplotype; WEffect is the predicted effect of the haplotype on the phenotype calculated using summation of weighted effects of each markers; Effect is the categorical effect of the haplotype where positive homozygous marker has an effect of +1, negative homozygous marker has an effect of −1, and heterozygous marker has half of the effect of the homozygous one. SNP_ID is the list of the IDs of all the top-ranking markers of the haplotype, separated by “|”. Within the column Lines, “BEST” represents the haplotype predicted to have the most significant positive effect on phenotype and “WORST” represents the haplotype predicted to have the most significant negative effect.

Supplementary File 4 All the predicted haplotypes for each of the 10 key haplotype blocks and their mapping to all genotyped barley lines associated with flowering time. Each sheet of the Excel file represents one haplotype block. Hap is the predicted haplotype; Lines are the barley lines that have the same haplotype where each line is separated by “|”; Line_Count is the number of lines that have the same haplotype; WEffect is the predicted effect of the haplotype on the phenotype calculated using summation of weighted effects of each markers; Effect is the categorical effect of the haplotype where positive homozygous marker has an effect of +1, negative homozygous marker has an effect of −1, and heterozygous marker has half of the effect of the homozygous one. SNP_ID is the list of the IDs of all the top-ranking markers of the haplotype, separated by “|”. Within the column Lines, “BEST” represents the haplotype predicted to have the most significant positive effect on phenotype and “WORST” represents the haplotype predicted to have the most significant negative effect.
