# Supplementary material for: Machine learning to predict genotypes and genotype-environment interaction associated with complex traits for genomic selection
Source: Plant Phenomics. 2026 May 19;8(3):100224. doi: 10.1016/j.plaphe.2026.100224 (PMC13320483; doi:10.1016/j.plaphe.2026.100224)
Supplement: Multimedia component 1 [file mmc1.pdf]

Table S1. The BOM weather stations used for collecting the weather data.

Trial Site	Station Number	Latitude	Longitude
**Geraldton**	008315	28.80° S	114.70° E
**Meredin**	010092	31.48° S	118.28° E
**South Perth**	009225	31.92° S	115.87° E
**Katannin**	010916	33.69° S	117.61° E
**Esperance**	009542	33.68° S	121.83° E

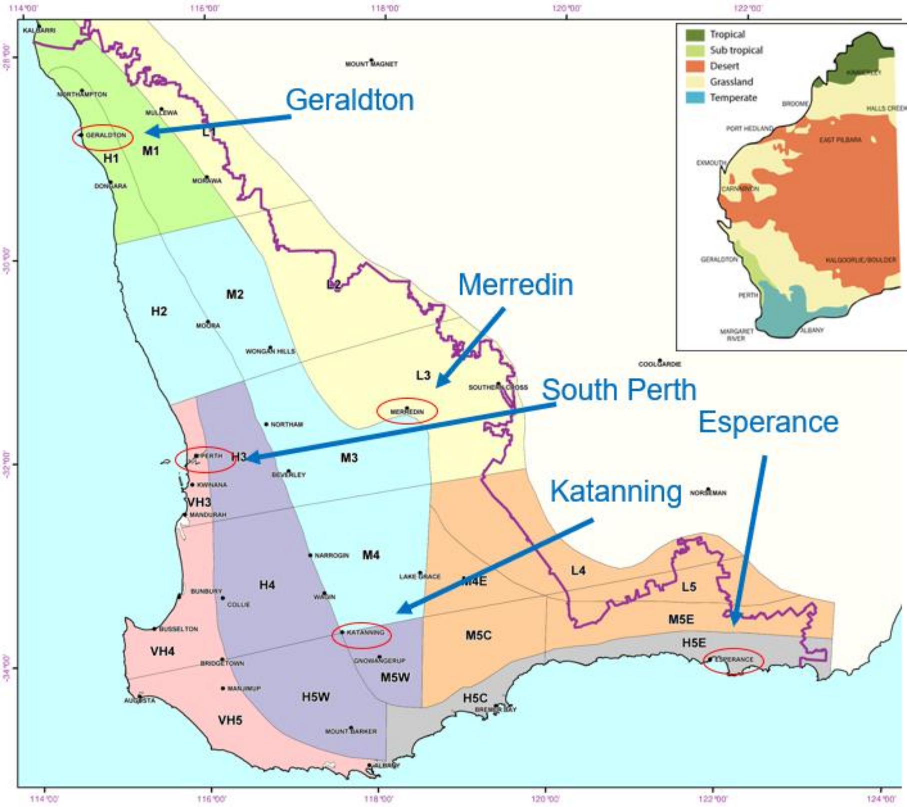

Fig. S1. Map of the five barley field trial sites across Western Australia, with trial locations projected against agricultural zones; climatic differences across these regions provide environmental context for phenotypic variation.

[OFFICIAL]

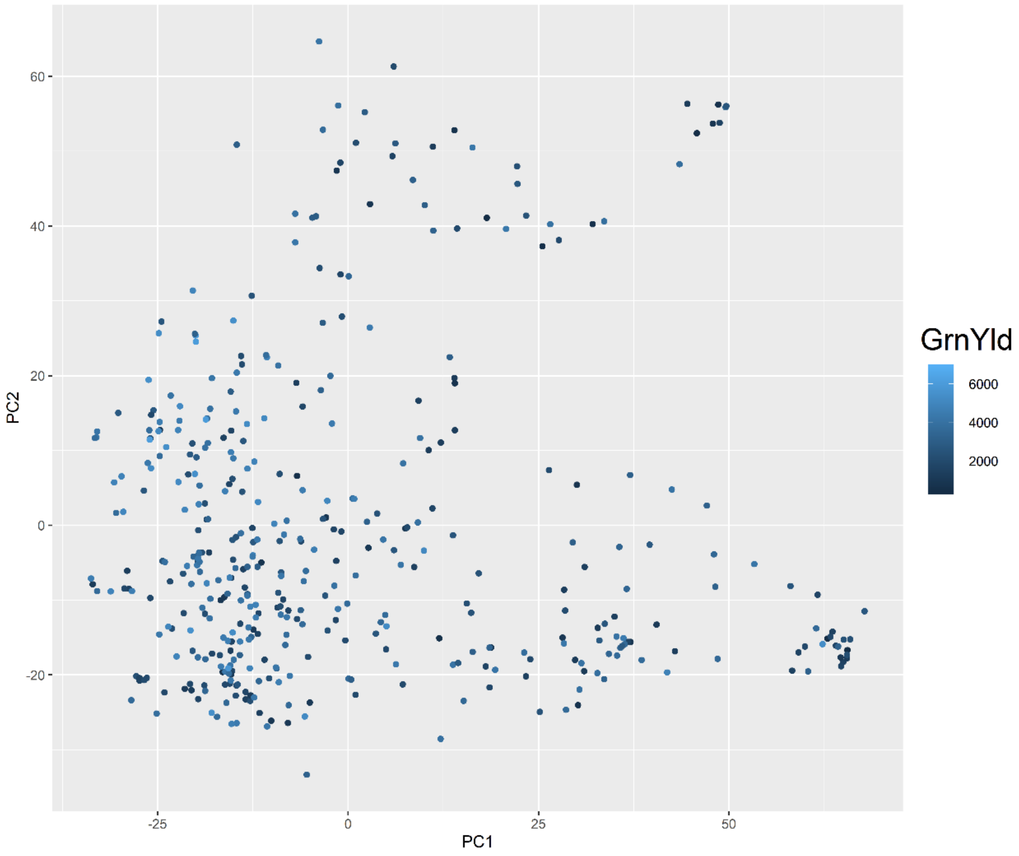

Fig. S2. Principal Components Analysis (PCA) of 855 barley lines based on genome-wide markers. Each dot represents a line and is coloured by grain yield (kg/ha) on a blue gradient, with darker blue indicating lower yield. X axis: first principal component; Y axis: second principal component.

[OFFICIAL]

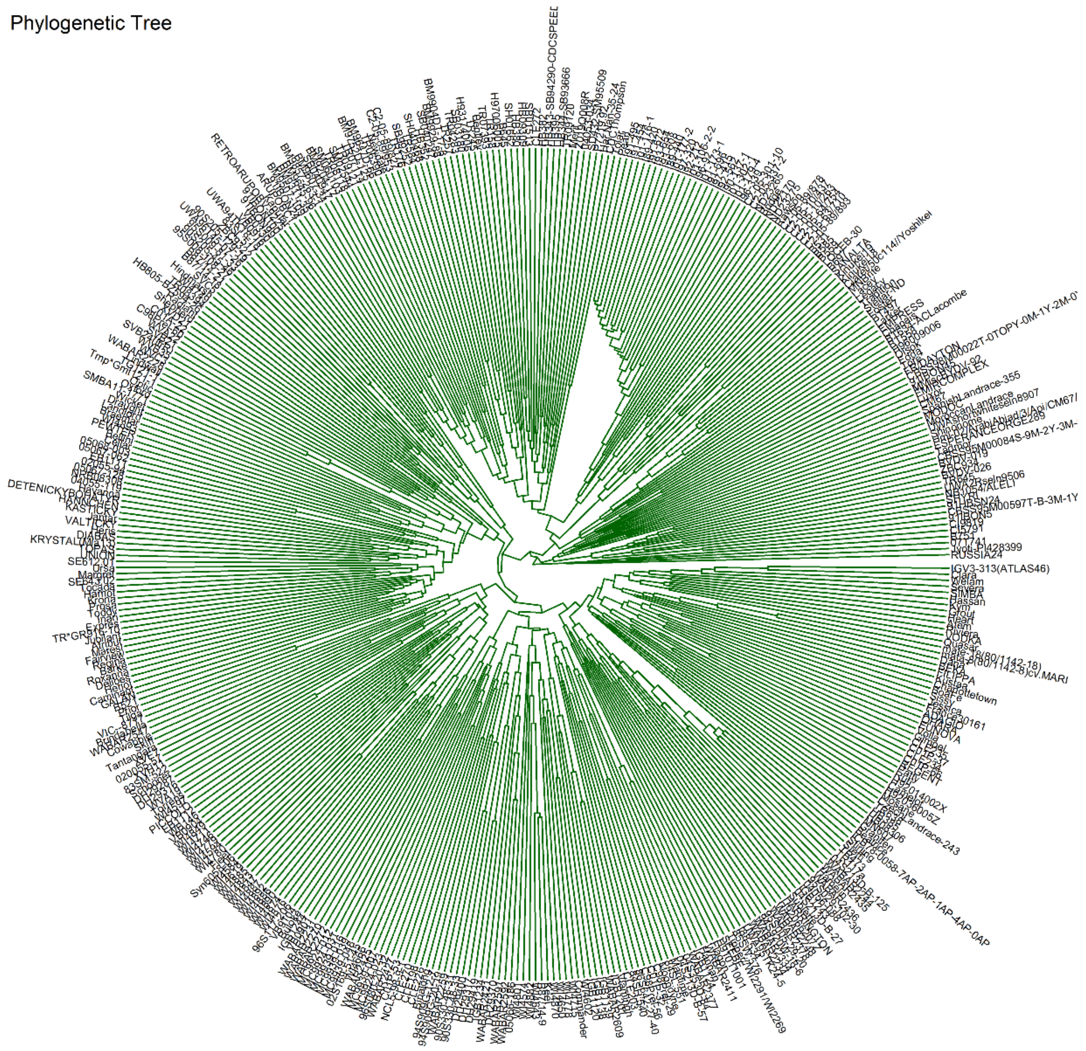

Fig. S3. The phylogenetic tree of the 855 barley varieties included in the modelling. The tree was generated by using neighbour-joining on the distance matrix generated by all the markers. The tree is visualised as unrooted tree.

[OFFICIAL]

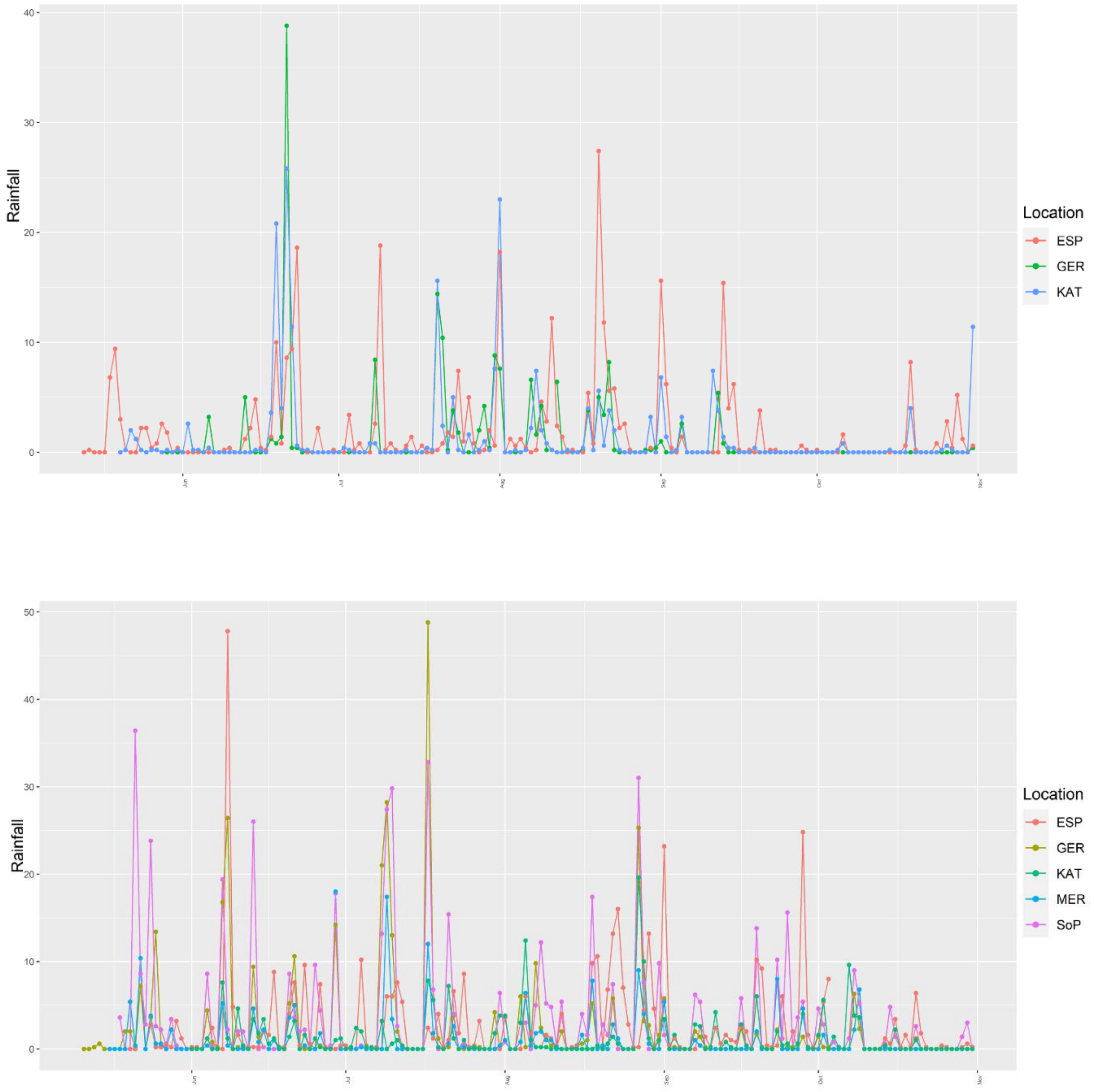

Fig. S4. Daily rainfall (mm) from sowing until harvest for the 2015 and 2016 field trials, collected from the Bureau of Meteorology (BOM). Time series spans planting to harvest dates for each environment. Lines are coloured by site: ESP (Esperance), GER (Geraldton), KAT (Katanning), MER (Merredin), SoP (South Perth).

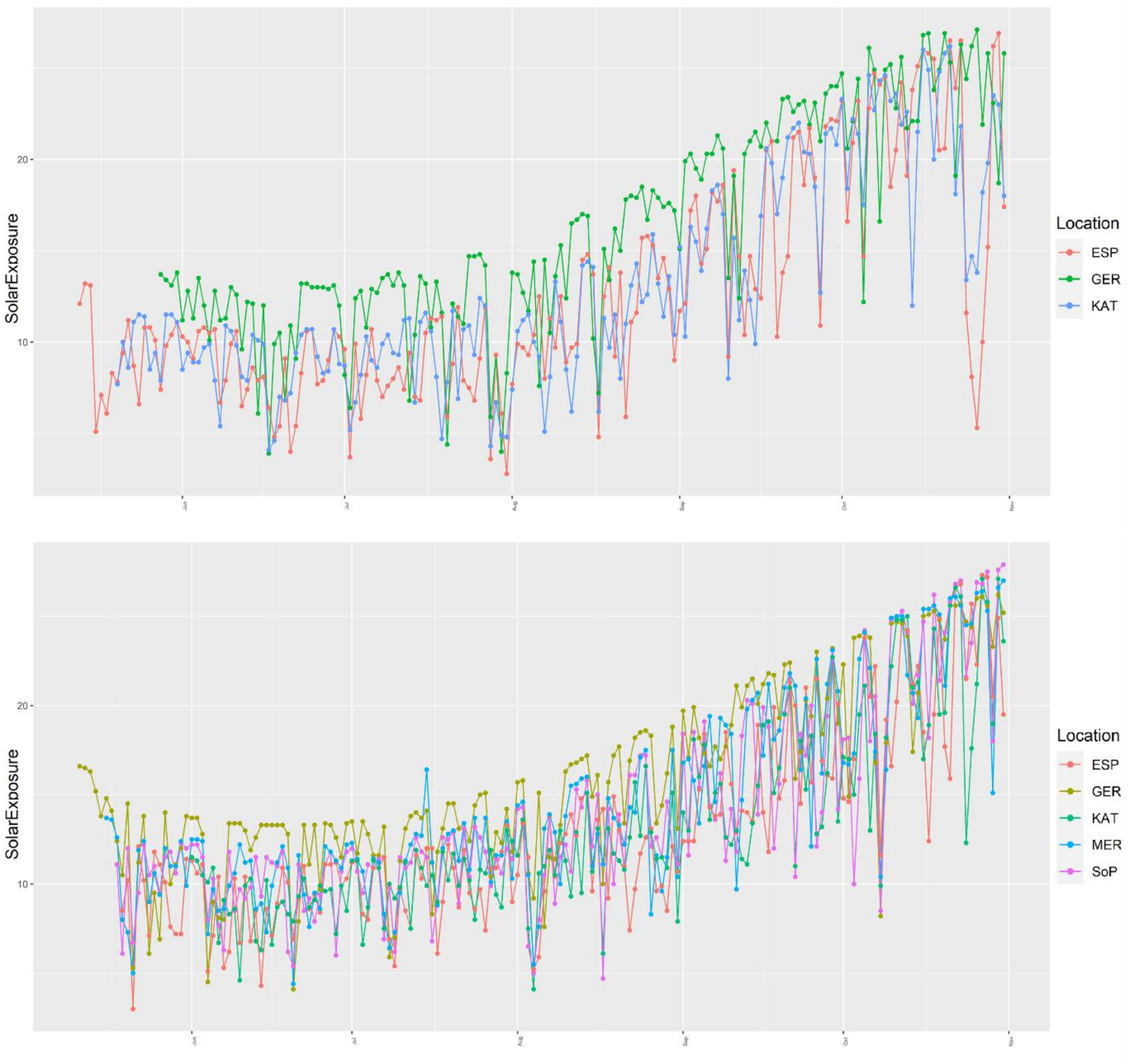

Fig. S5. Daily solar exposure (MJ m^{-2}) across the growing season for 2015 and 2016 trials, showing temporal patterns from planting to harvest. Each coloured line corresponds to a trial site: ESP (Esperance), GER (Geraldton), KAT (Katanning), MER (Merredin), SoP (South Perth).

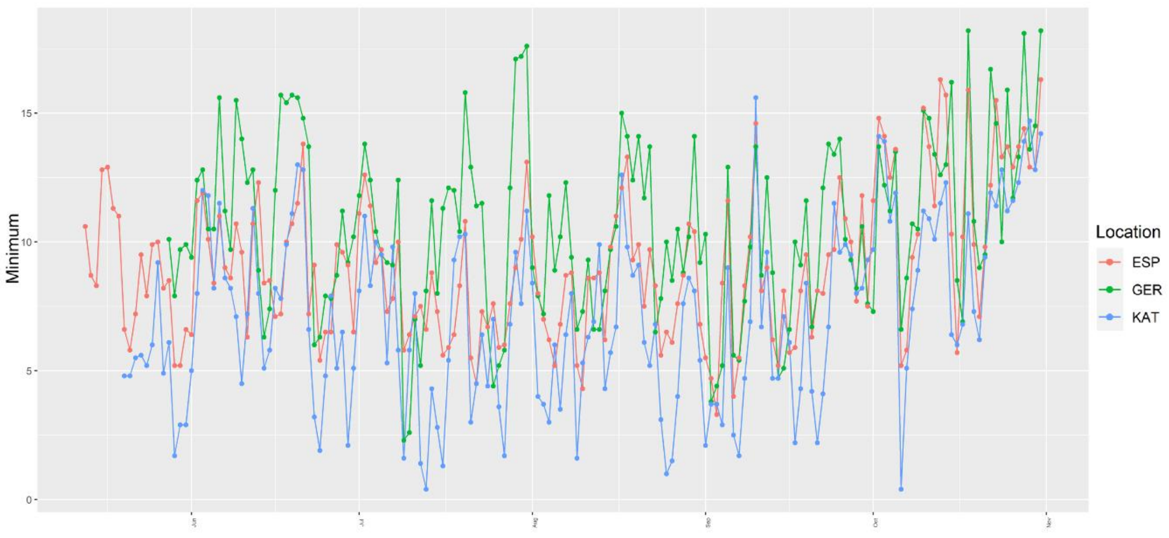

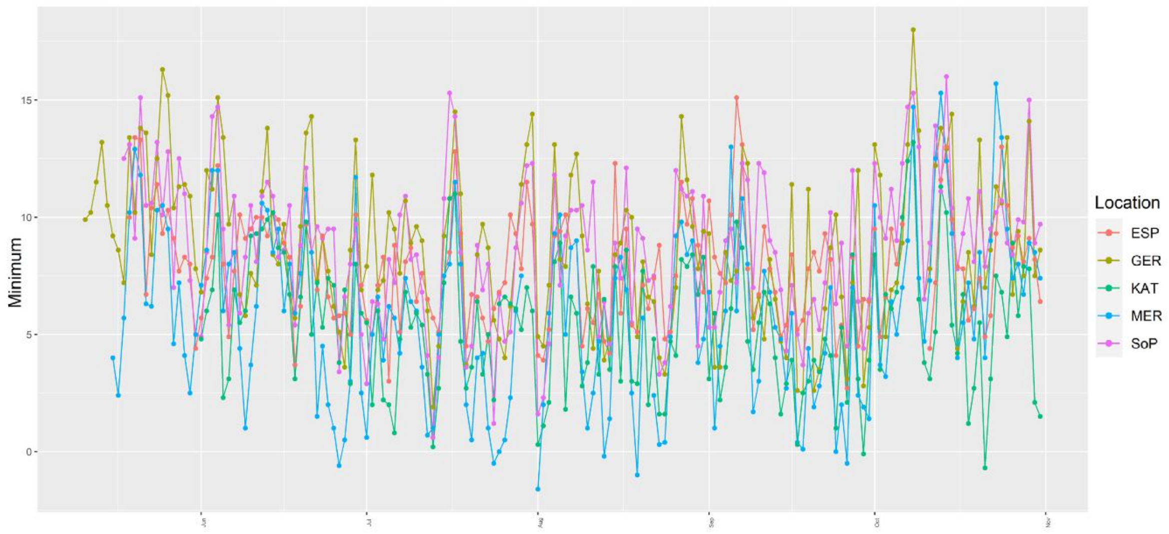

Fig. S6. The daily minimum temperature across the growing season collected from BOM of 2015 and 2016 respectively. X axis is date, and Y axis is the minimum temperature (C). ESP represents Esperance; GER represents Geraldton; KAT represents Katanning; MER represents Merredin; and SoP represents South Perth.

[OFFICIAL]

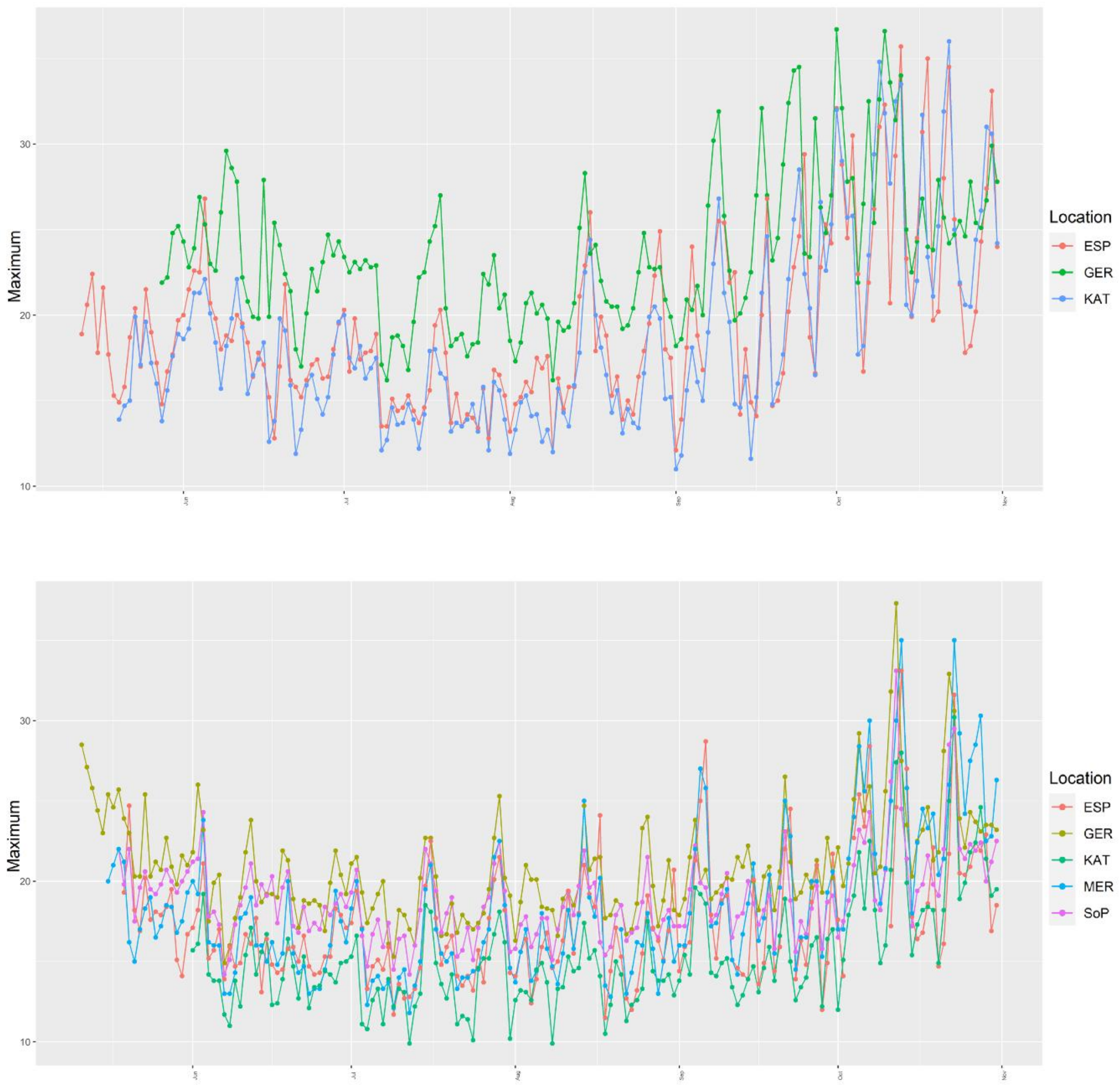

Fig. S7. The daily maximum temperature across the growing season collected from BOM of 2015 and 2016 respectively. X axis is date, and Y axis is the maximum temperature (C). ESP represents Esperance; GER represents Geraldton; KAT represents Katanning; MER represents Merredin; and SoP represents South Perth.

[OFFICIAL]

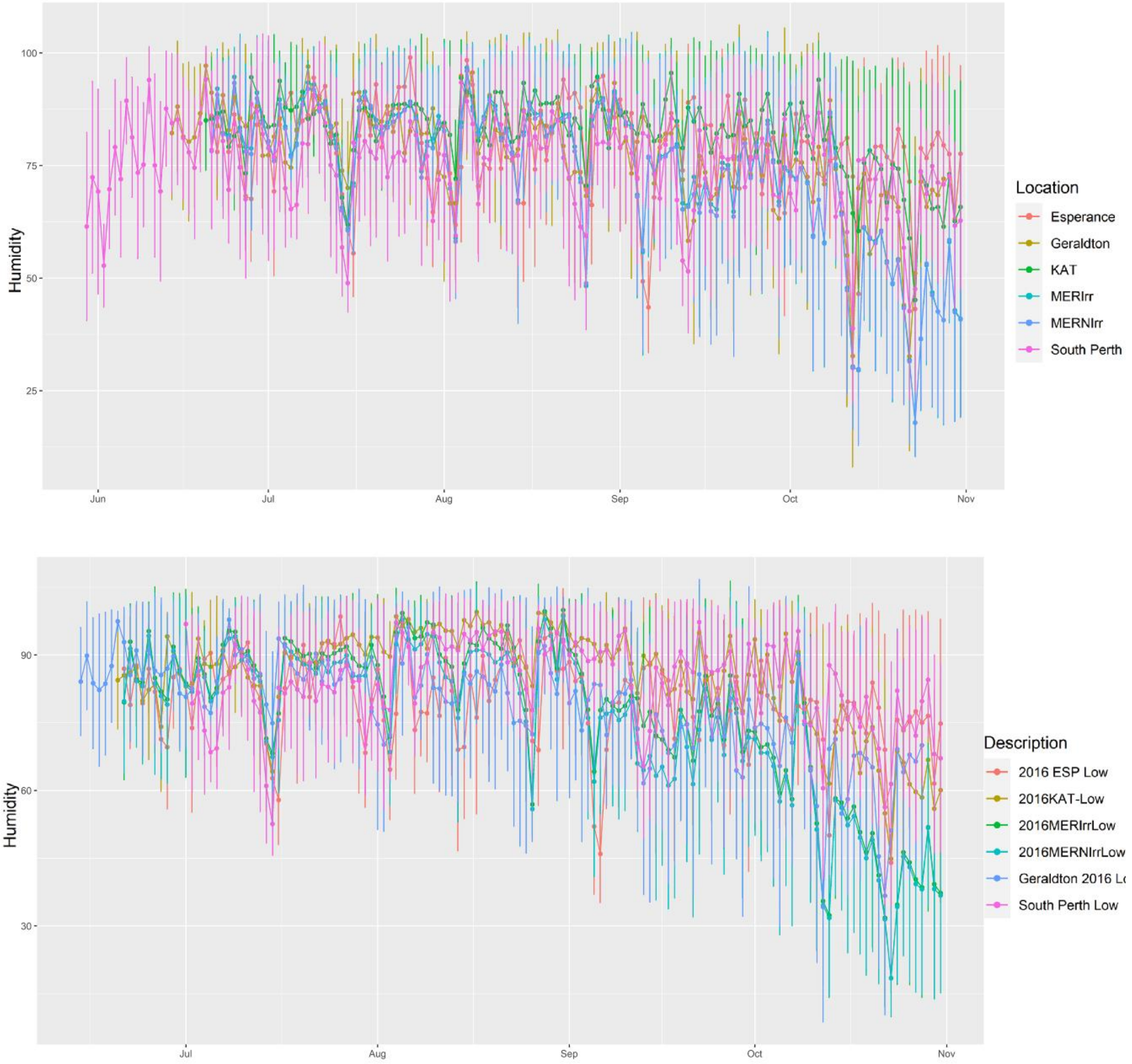

Fig. S8 The humidity of the field sites every 15 minutes measured by the sensors installed in the air and near the canopy of the crop from date of planting to harvest. X axis is date, and Y axis is the humidity (%). The upper subplot is the humidity 20 cm above the plants while the lower subplot is the measurements at canopy. ESP represents Esperance; GER represents Geraldton; KAT represents Katanning; MER represents Merredin; and SoP represents South Perth.

[OFFICIAL]

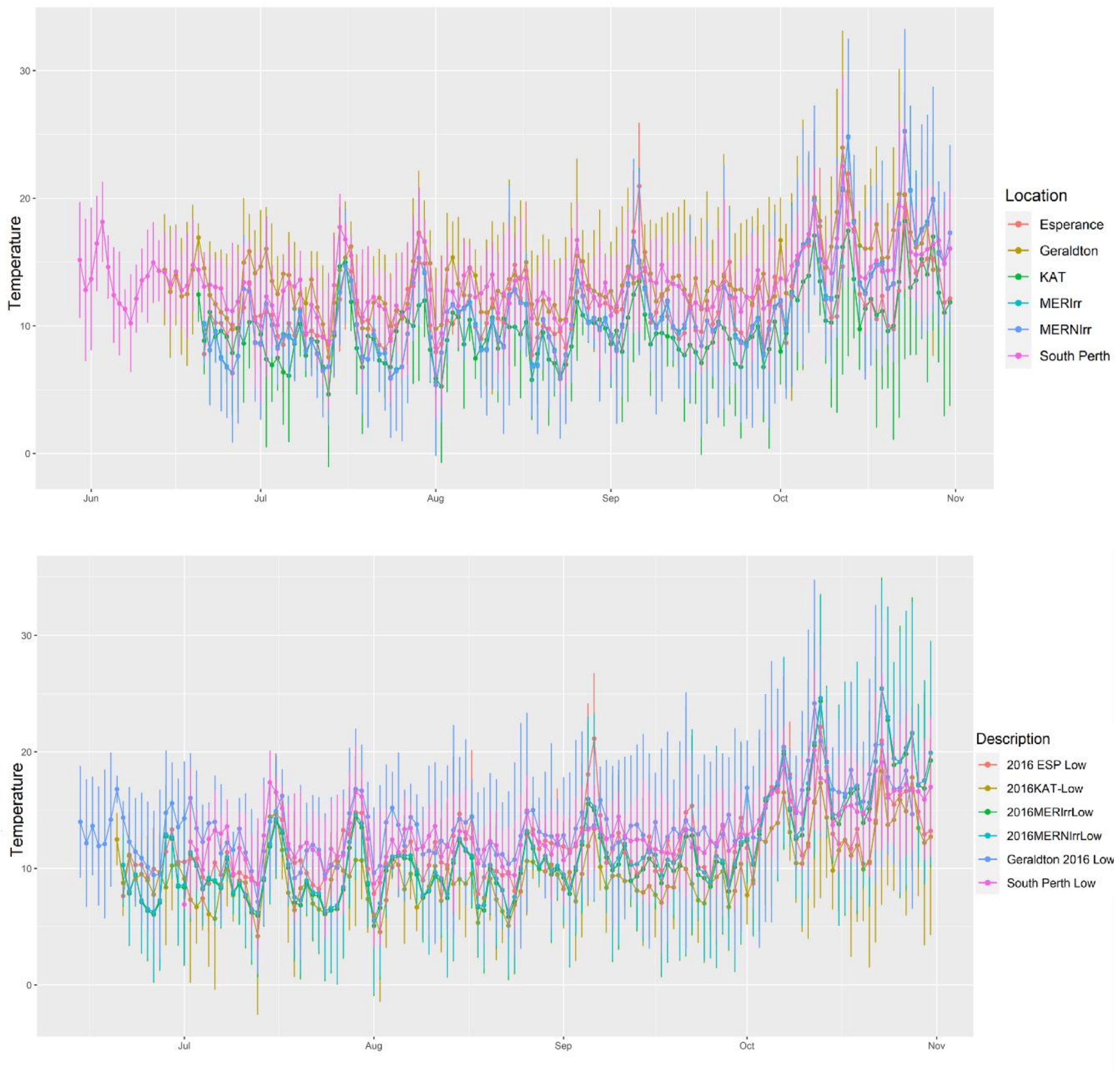

Fig. S9. The temperature of the field sites every 15 minutes measured by the sensors installed in the air and near the canopy of the crop from date of planting to harvest. X axis is date, and Y axis is the temperature (C). The upper subplot is the temperature 20 cm above the plants while the lower subplot is the measurements at canopy. ESP represents Esperance; GER represents Geraldton; KAT represents Katanning; MER represents Merredin; and SoP represents South Perth.

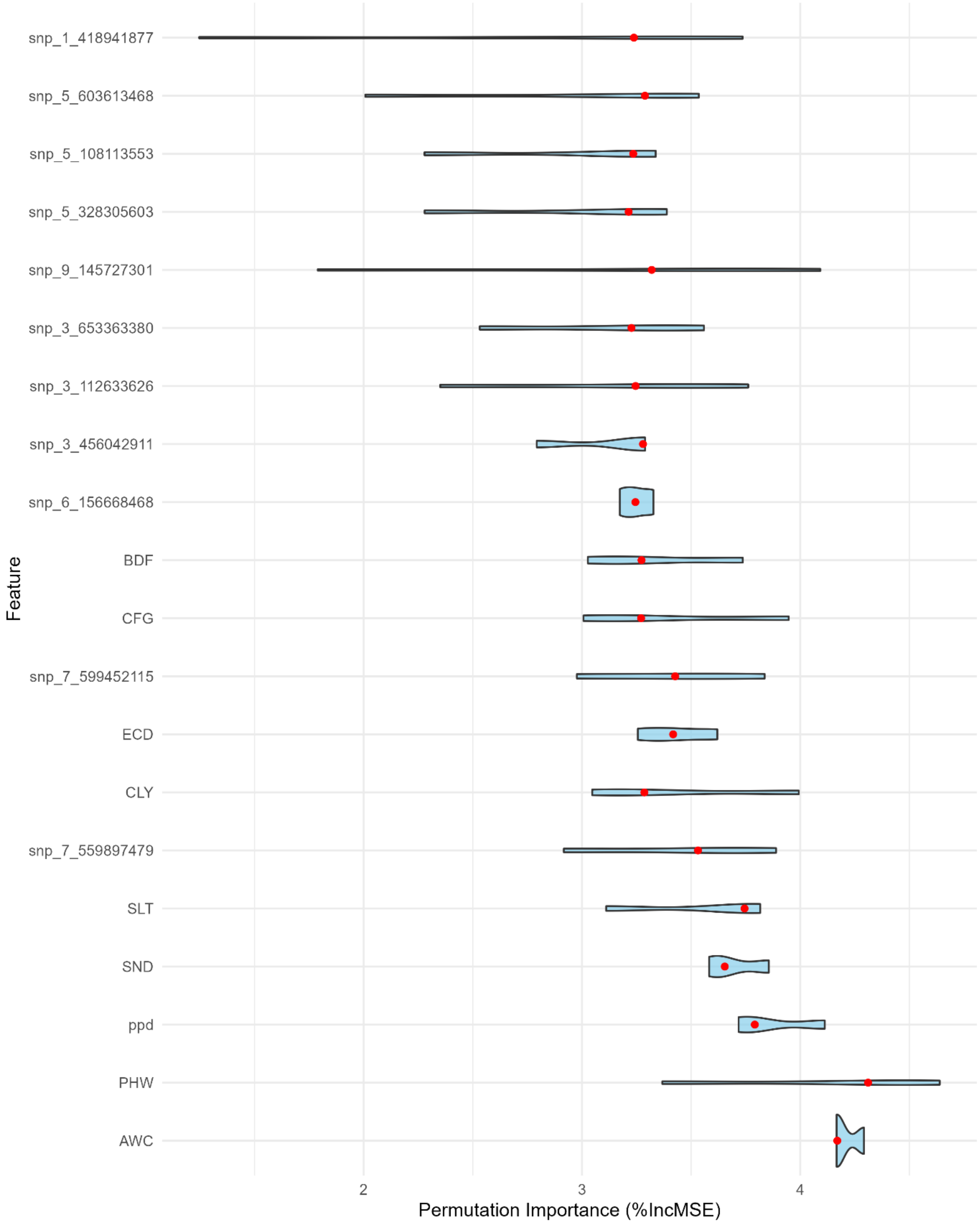

Fig. S10. Violin plots showing stability (distribution) of key environmental and soil features selected across repeated recursive feature elimination (RFE) runs. Each violin represents the distribution of selection frequency or importance score for a feature across cross-validation folds. Features are annotated with measurement units and temporal aggregation method.

[OFFICIAL]

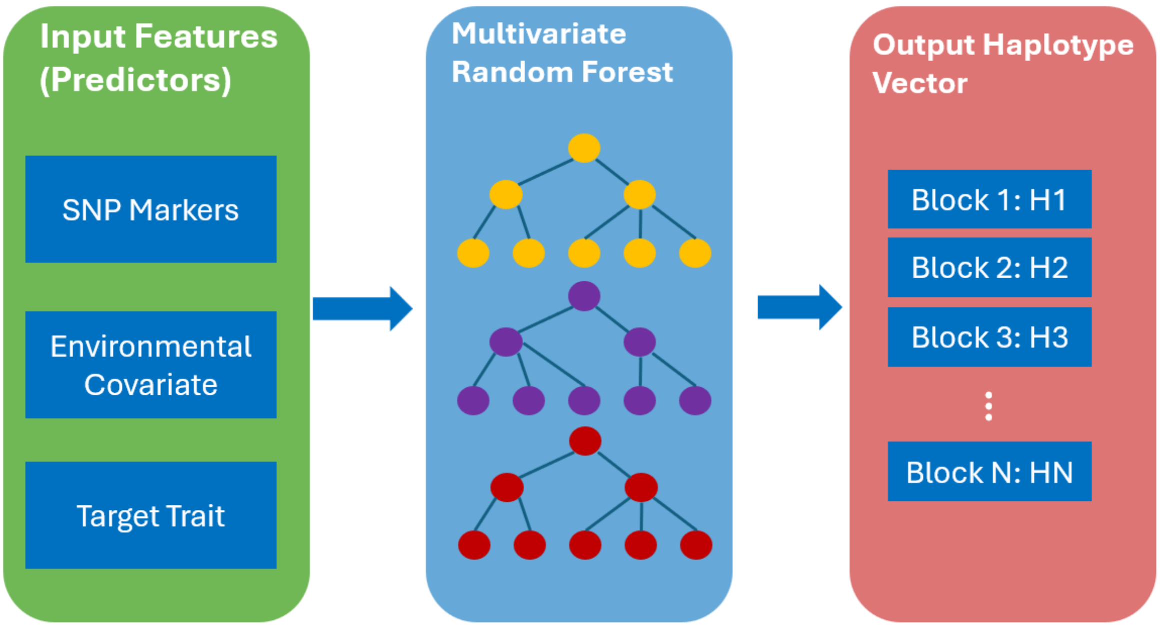

Fig. S11. Conceptual illustration of the multivariate random forest used for haplotype prediction. Each input sample consists of genotype markers, environmental covariates, and a specified target phenotypic value. These predictors are input into a multivariate random forest composed of many decision trees, each trained on bootstrap resamples. The forest jointly predicts a vector of haplotype block assignments, one predicted haplotype per block, which together form the output haplotype profile for a given target trait and environment. This schematic emphasises that the model simultaneously estimates multiple categorical responses (haplotype blocks) in a single ensemble, leveraging correlations among blocks to improve predictive performance.

Table S2. The soil parameters of the trial sites. GER: Geraldton, ESP: Esperance, KAT: Katanning, MER: Merredin, STH PER: South Perth.

**Location**	**Latitude**	**Longitude**	**AWC**	**BDF**	**BDW**	**CLY**	**CFG**	**ECD**	**PHW**	**SND**	**SLT**
**GER**	-28.7784	114.6572	8.168736	1.38152	1.3988	10.36311	2.488356	0.059369	6.27708	87.13006	2.505801
**ESP**	-33.609	121.7734	4.797651	1.428268	1.478251	4.546297	6.781714	0.078399	5.829806	94.35396	1.099295
**KAT**	-33.6883	117.6343	5.793028	1.433886	1.494296	8.289745	7.954996	0.217459	5.867478	89.70497	2.00446
**MER**	-31.5057	118.2243	10.00534	1.429486	1.446331	14.66943	2.52135	0.120012	6.460129	81.78204	3.547069
**STHPER**	-31.991	115.8875	4.038568	1.425207	1.508257	3.733778	9.003229	0.066083	7.155143	95.36302	0.902828

[OFFICIAL]

Table S3. The prediction results of grain yield over different evaluation methods, including Leave-one-site-out, Leave-one-line-out, and five-fold cross validation.

**Leave-Line-Out**	**RMSE**	**R^2**	**MAE**	**RMSE SD**	**R^2 SD**	**MAE SD**
RF	0.609849	0.515954	0.475831	0.043554	0.044866	0.030991
GxE	0.610535	0.617026	0.463684	0.044783	0.034838	0.038742
G+E	0.634628	0.584609	0.467366	0.040557	0.033314	0.03658
G	0.741883	0.431065	0.557035	0.059886	0.061034	0.044832
**5 Fold CV**	**RMSE**	**R^2**	**MAE**	**RMSE SD**	**R^2 SD**	**MAE SD**
RF	0.429542	0.813036	0.293491	0.022898	0.018072	0.010588
GxE	0.668417	0.668417	0.517217	0.007932	0.007932	0.008538
G+E	0.676951	0.542967	0.522799	0.007386	0.017286	0.007383
G	0.800026	0.362019	0.626956	0.010908	0.031255	0.013714
**Leave-Site-Out**	**RMSE**	**R^2**	**MAE**	**RMSE SD**	**R^2 SD**	**MAE SD**
RF	0.609849	0.515954	0.475831	0.043554	0.044866	0.030991
GxE	0.878504	0.489611	0.697335	0.412885	0.102981	0.312334
G+E	1.106921	0.20709	0.964129	0.463168	0.003458	0.457473
G	0.817107	0.210392	0.676899	0.000905	0.000911	0.000795

Table S4. The prediction results of flowering time (ZS49) over different evaluation methods, including Leave-one-site-out, Leave-one-line-out, and five-fold cross validation.

**Leave-Line-Out**	**RMSE**	**R^2**	**MAE**	**RMSE SD**	**R^2 SD**	**MAE SD**
RF	0.486714	0.751372	0.383951	0.138133	0.111658	0.082102
GxE	0.451182	0.788888	0.305303	0.079457	0.082618	0.046338
G+E	0.487608	0.759502	0.316563	0.069494	0.066445	0.046641
G	0.785935	0.356987	0.629521	0.071126	0.05011	0.047567
5 Fold CV	**RMSE**	**R^2**	**MAE**	**RMSE SD**	**R^2 SD**	**MAE SD**
RF	0.69046	0.524562	0.530026	0.015597	0.023899	0.020011
GxE	0.42146	0.821646	0.288825	0.016763	0.012242	0.007709
G+E	0.427141	0.816853	0.291648	0.017915	0.012701	0.008286
G	0.829054	0.309302	0.669363	0.016676	0.022517	0.014351
Leave-Site-Out	**RMSE**	**R^2**	**MAE**	**RMSE SD**	**R^2 SD**	**MAE SD**
RF	0.345352	0.488728	0.242128	0.026305	0.049284	0.007648
GxE	1.008136	0.550514	0.955277	0.198426	0.060249	0.210692
G+E	0.959057	0.424726	0.886436	0.432344	0.112657	0.464168
G	0.810568	0.282183	0.700689	0.131742	0.13069	0.126564

Illustrative Workflow for the Shiny App

Objective: Demonstrate how a user can interact with the Shiny app to generate haplotype predictions for a target phenotype under specified environmental conditions.

[OFFICIAL]

Step 1: Launch and Select Task: Open the Shiny application in a web browser (link provided in the main text). On the landing page, choose either "Predicting Phenotypes from Genotype x Environment" or "Predicting Genotype from Phenotype x Environment" from the left panel.

Step 2: Specify Target Phenotype and Environment: In the input panel, select "How many % used for testing prediction" which is the training and testing split percentage, "Choose a trait to predict" (Flowering time or Grain yield), "Choose years of trial data to be used" (2015, 2016 or 2015 and 2016) which select which year's data to be used in the prediction.

Step 3: Submit and Visualise: Click "Start Prediction Now". The app will start modelling and displays:

- A scatter plot to demonstrate the predicted values and the true values.
- A Pearson correlation between the predicted values and the true values.
- The list of the predicted and true values.

Step 4: Interpretation and Export: Users can download the model results into local drive for other and downstream analysis.

[OFFICIAL]
